# Stimulating leisure-time activities and the risk of dementia: a multi-cohort study

**DOI:** 10.1093/ageing/afae141

**Published:** 2024-07-13

**Authors:** Katriina Heikkilä, Jaana Pentti, Serhiy Dekhtyar, Jenni Ervasti, Laura Fratiglioni, Tommi Härkänen, Mika Kivimäki, Seppo Koskinen, Tiia Ngandu, Säde Stenlund, Sakari Suominen, Jussi Vahtera, Suvi Rovio, Sari Stenholm

**Affiliations:** Department of Public Health, University of Turku and Turku University Hospital, Kiinamyllynkatu 10, 20520 Turku, Finland; Centre for Population Health Research, University of Turku and Turku University Hospital, Kiinamyllynkatu 10, 20520 Turku, Finland; Department of Public Health and Welfare, Finnish Institute for Health and Welfare, Mannerheimintie 166, 00300 Helsinki, Finland; Department of Public Health, University of Turku and Turku University Hospital, Kiinamyllynkatu 10, 20520 Turku, Finland; Centre for Population Health Research, University of Turku and Turku University Hospital, Kiinamyllynkatu 10, 20520 Turku, Finland; Clinicum, Faculty of Medicine, University of Helsinki, Tukholmankatu 8 B, 00014 Helsinki, Finland; Aging Research Centre, Department of Neurobiology, Care Sciences and Society, Karolinska Institutet and Stockholm University, 171 65 Solna Stockholm, Sweden; Stockholm Gerontology Research Center, Sveavägen 155, 113 46 Stockholm, Sweden; Finnish Institute of Occupational Health, PB 40, 00032 Helsinki, Finland; Aging Research Centre, Department of Neurobiology, Care Sciences and Society, Karolinska Institutet and Stockholm University, 171 65 Solna Stockholm, Sweden; Stockholm Gerontology Research Center, Sveavägen 155, 113 46 Stockholm, Sweden; Department of Public Health and Welfare, Finnish Institute for Health and Welfare, Mannerheimintie 166, 00300 Helsinki, Finland; Clinicum, Faculty of Medicine, University of Helsinki, Tukholmankatu 8 B, 00014 Helsinki, Finland; Finnish Institute of Occupational Health, PB 40, 00032 Helsinki, Finland; UCL Faculty of Brain Sciences, University College London, Gower Street, London WC1E 6BT, UK; Department of Public Health and Welfare, Finnish Institute for Health and Welfare, Mannerheimintie 166, 00300 Helsinki, Finland; Department of Public Health and Welfare, Finnish Institute for Health and Welfare, Mannerheimintie 166, 00300 Helsinki, Finland; Stockholm Gerontology Research Center, Sveavägen 155, 113 46 Stockholm, Sweden; Department of Public Health, University of Turku and Turku University Hospital, Kiinamyllynkatu 10, 20520 Turku, Finland; School of Population and Public Health, University of British Columbia, 2206 E Mall, Vancouver, BC V6T 1Z3, Canada; Department of Public Health, University of Turku and Turku University Hospital, Kiinamyllynkatu 10, 20520 Turku, Finland; School of Health Sciences, University of Skövde, Högskolevägen, Box 408541 28, Skövde, Sweden; Department of Public Health, University of Turku and Turku University Hospital, Kiinamyllynkatu 10, 20520 Turku, Finland; Centre for Population Health Research, University of Turku and Turku University Hospital, Kiinamyllynkatu 10, 20520 Turku, Finland; Department of Public Health, University of Turku and Turku University Hospital, Kiinamyllynkatu 10, 20520 Turku, Finland; Centre for Population Health Research, University of Turku and Turku University Hospital, Kiinamyllynkatu 10, 20520 Turku, Finland; Research Centre of Applied and Preventive Cardiovascular Medicine, University of Turku, Kiinamyllynkatu 10, 20520 Turku, Finland; Department of Public Health, University of Turku and Turku University Hospital, Kiinamyllynkatu 10, 20520 Turku, Finland; Centre for Population Health Research, University of Turku and Turku University Hospital, Kiinamyllynkatu 10, 20520 Turku, Finland; Research Services, Turku University Hospital and University of Turku, Kiinamyllynkatu 4-8, 20520 Turku, Finland

**Keywords:** dementia, activity, cognitive, prospective study, meta-analysis, older people

## Abstract

**Background:**

Stimulating activities are associated with a decreased risk of dementia. However, the extent to which this reflects a protective effect of activity or non-participation resulting from dementia is debated. We investigated the association of stimulating leisure-time activity in late adulthood with the risk of dementia across up to two decades’ follow-up.

**Methods:**

We used data from five prospective cohort studies from Finland and Sweden. Mental, social, outdoor, consumptive and physical leisure-time activities were self-reported. Incident dementia was ascertained from clinical diagnoses or healthcare and death registers. Cox regression was used to estimate hazard ratios (HRs) and 95% confidence intervals (CIs).

**Results:**

Of the 33 263 dementia-free individuals aged ≥50 years at baseline, 1408 had dementia during a mean follow-up of 7.0 years. Active participation in mental (HR: 0.52, 95% CI: 0.41 to 0.65), social (HR: 0.56 95% CI: 0.46 to 0.72), outdoor (HR: 0.70, 95% CI: 0.58 to 0.85), consumptive (HR: 0.67, 95% CI: 0.53 to 0.94) and physical (HR: 0.62, 95% CI: 0.51 to 0.75) activity, as well as variety (HR: 0.54, 95% CI: 0.43 to 0.68) and the overall frequency of activity (HR: 0.41, 95% CI: 0.34 to 0.49) were associated with a reduced risk of dementia in <10 years’ follow-up. In ≥10 years’ follow-up all associations attenuated toward the null.

**Conclusion:**

Stimulating leisure-time activities are associated with a reduced risk of dementia in short-term but not long-term follow-up. These findings may reflect a reduction in leisure-time activity following preclinical dementia or dilution of the association over time.

## Key Points

- We examined the association of stimulating leisure-time activities in late adulthood with the risk of dementia.
- Active leisure-time participation was associated with a decreased risk in <10 years’ follow-up.
- These associations attenuated to null when the follow-up extended beyond 10 years.
- Our findings may reflect a dilution of the effect over time or reverse causality.

## Introduction

Dementia affects an estimated 57 million people worldwide and it is one of the leading causes of disability and death for older people worldwide [1, 2]. The increasing prevalence of dementia, linked to population ageing, poses enormous challenges to healthcare systems in providing care for older adults experiencing cognitive and functional decline [1]. In Europe and the UK, the currently available medical treatments for the main types of dementia, such as Alzheimer’s disease, are not disease-modifying, and novel agents available in countries such as the USA may offer some disease-modifying effects but can also present side effects [3]. Thus, efforts must focus on prevention and delaying the onset of symptoms. Public health guidelines in many countries recommend cognitively and socially stimulating activities as a means of preventing or delaying the onset of dementia [4–6].

A clinical diagnosis of dementia is typically preceded by a long subclinical phase, which may present opportunities to detect risk factors indicative of the early stages of the disease process, as well as resilience factors potentially slowing down this process [7]. Cognitive stimulation has been hypothesised to increase resilience to dementia pathology, via its neuroprotective and compensatory influences on brain function [8]. These may manifest as lesser accumulation of dementia-associated brain lesions, an augmented neuroanatomical resource that provides surplus capacity to maintain cognitive function despite accumulating brain pathology (i.e. brain reserve), or an enhanced flexibility in brain function that helps stave off symptoms of degenerative brain changes associated with dementia (i.e. cognitive reserve) [8]. Engagement in cognitively, socially and physically stimulating activities throughout life has been highlighted as a way of cultivating resilience and participation in leisure-time activities could be a means for doing this [8]. Leisure-time activity participation, such as many other dementia risk factors, is socioeconomically patterned, and the overall amount, diversity and types of specific stimulating activities people undertake vary across cultures and contexts.

Epidemiological evidence suggests that older people who regularly engage in cognitively stimulating leisure-time activities (i.e. activities that involve information seeking or processing) are less likely to develop dementia than their counterparts who are less cognitively active [9, 10]. However, many studies I have examined a limited number of activities and had relatively short follow-up periods (~2–7 years) and few studies have examined the temporality of the association beyond this timescale [9, 11–15]. Findings from studies with long-term follow-up have had inconclusive findings, with positive and null-associations reported across varying follow-up periods [13–15].

The aim of our investigation was to examine the association of stimulating leisure-time activities (specific activity domains as well as variety and the overall frequency of activity) in late adulthood with the risk of dementia across two decades of follow-up. To do this, we utilised data from five independent cohort studies, pooling the estimates in meta-analyses.

## Methods

### Study population

**Figure 1:**
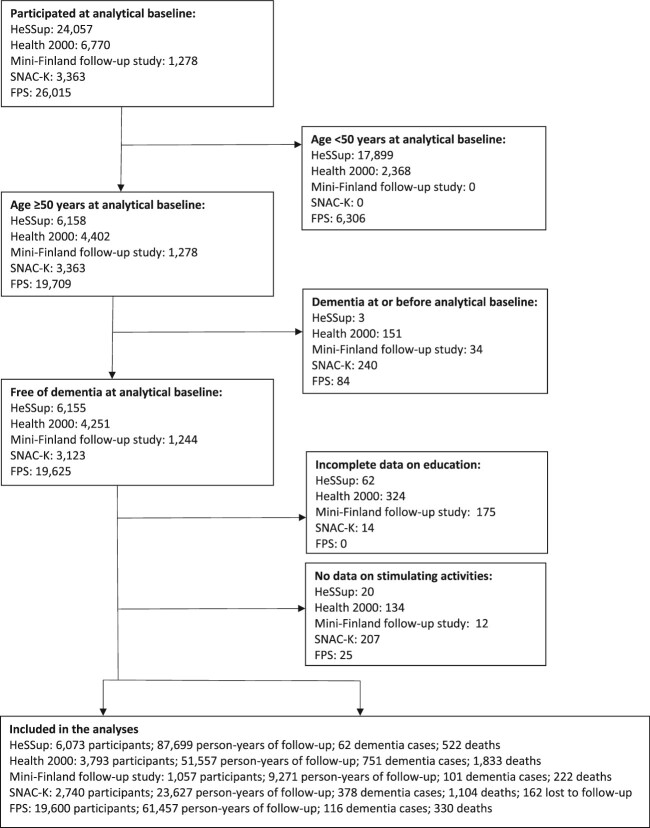
Participant flow chart.

We used individual-level data from five prospective cohort studies: Health and Social Support (HeSSup), Health 2000, Mini-Finland Follow-up Study and Finnish Public Sector study (FPS) from Finland, and Swedish National Study on Aging and Care in Kungsholmen (SNAC-K) from Sweden. Details of the studies’ design and data collection are provided in Supplementary data. Briefly, HeSSup, Health 2000, Mini-Finland Follow-up study and SNAC-K are population-based studies and FPS is an occupational cohort. Our analyses in all studies were based on data from participants who were aged ≥50 years at the time when stimulating leisure-time activities were ascertained and who had available data on at least one activity, age, sex and education and no record of dementia during or before the baseline year (Figure 1).

### Stimulating leisure-time activities

Leisure-time activities were self-reported at baseline: 1998 in HeSSup, 2000–2001 in Health 2000 and Mini-Finland Follow-up Study, 2001–2004 in SNAC-K and 2013 in FPS. Activities were categorised into five domains: mental (e.g. reading and studying), social (e.g. participating in clubs and visiting with friends or relatives), outdoor (e.g. hunting and gardening), consumptive (e.g. going to the cinema, theatre and concerts) and physical activity (e.g. walking, jogging and exercise) (Supplementary data). Participants indicated the frequency of taking part in each activity on Likert-type scales, with the response options ranging from daily or most days to never. The mean response for each activity domain was calculated for each participant and the study-specific mean scores were divided into quartiles; participants whose score was in the highest quartile in each study were defined as active participants in the relevant activity domain. In addition, we calculated two overall measures of activity: variety and overall frequency. The variety of activity was operationalised as the number of activity domains in which a participant’s mean score was in the highest quartile of participation, and the overall frequency of activity was operationalised as the participant’s highest activity participation quartile across the five domains, regardless of how many domains were involved.

### Dementia

In the Finnish studies, dementia was ascertained from nationwide hospital care, medication purchase or reimbursement, and causes of death registers (Supplementary data). In SNAC-K dementia ascertained from structured interviews, clinical examination, cognitive testing and physician review of medical records and death certificates (Supplementary data).

### Covariates

Covariates were measured at baseline and are described in detail in Supplementary data. Briefly, age and sex were ascertained from Statistics Finland population data in Mini-Finland Follow-up Study, Health 2000 and HeSSup, and from the employer’s records in FPS. Achieved education, an indicator of socioeconomic position and an important predictor of dementia risk [16, 17], was ascertained from baseline self-reporting questionnaire in HeSSup, Health 2000 and Mini-Finland Follow-up Study and from Statistics Finland data in FPS. In SNAC-K, age, sex and education were self-reported. Education was categorised into basic (≤9 years), intermediate (10–12 years) or high (≥ 13 years). Depressive symptoms were ascertained from self-reported responses to Beck Depression Inventory [18] in HeSSup, Health 2000 and Mini-Finland (≥10 points indicating depressive symptoms), Montgomery-Åsberg depression rating scale [19] in SNAC-K (≥7 points indicating depressive symptoms) and questions on recently experienced depression, hopelessness or lack of interest in FPS. Mobility and sensory difficulties were ascertained from responses to questions on difficulties in walking 500 m [20], seeing or hearing, and analysed as binary variables (any difficulty vs. no difficulty) in all studies. *APOE* ε4 carrier status [21] in Health 2000 and SNAC-K was ascertained from blood samples genotyped using Illumina CoreExome, OMNIExpress and 610 K arrays in Health 2000 and a microsequencing method (AffiGen APOE; Sangtec Medical, Stockholm, Sweden) in SNAC-K.

### Statistical analyses

The association of leisure-time activities with incident dementia was examined using Cox proportional hazards regression (Supplementary data). The proportional hazards assumption was checked by inspecting log-(−log) plots. The timescale in the models was age in years [22]. The period under observation began on the date of activity ascertainment and ended on the first of the following: the date of dementia record, date of emigration from Finland or Sweden, date of death, the end of register follow-up (Finnish studies) or the date when the participant was last contacted (SNAC-K). We modelled the association of the activity exposures with dementia during two follow-up periods: <10 and ≥10 years [13]. Analyses with the follow-up periods split into <5, 5–9, 10–14 and ≥15 years were also conducted (Appendix 2). We examined multiplicative interaction with education by including an activity*education term in the main models. As the number of studies was small, we pooled study-specific estimates in fixed effects meta-analysis [23, 24].

We conducted five sets of sensitivity analyses: (i) using random effects meta-analyses with Sidik–Jonkman between-study variance estimator, which previous research suggests performs well with sparse data [23, 24]; (ii) restricting the study population to people aged ≥60 years at baseline; (iii) with additional adjustment for baseline depression, sensory difficulties, mobility difficulties and characteristics of the municipality of residence in a subset of studies with these data available; (iv) stratified by genetic risk (i.e. separately for individuals with any number of *APOE* ε4 alleles and those with no high-risk alleles) in Health 2000 and SNAC-K and (v) using an alternative, previously used categorisation of activities in SNAC-K (Supplementary data). All meta-analyses and the study-specific analyses in Health 2000, Mini-Finland Follow-up Study and SNAC-K were conducted using Stata SE 17 (Stata Corporation, College Station, TX, USA). The study-specific analyses in FPS and HeSSup were conducted using SAS 9.4 (SAS Institute, Cary, NC, USA).

## Results

In all, 34 201 women and men from the five cohort studies had no record of dementia at or before the analytical baseline. Individuals were included in the analyses if they had data on at least one stimulating leisure-time activity. We excluded individuals with no data on activities (*n* = 374, 1.1%) and those with incomplete data on education (*n* = 573, 1.7%) (Figure 1). Our analyses were thus based on data from 33 263 men and women, of whom 1408 (4.2%) had a record of dementia during a mean follow-up of 7.0 years (standard deviation: 1.7). The mean age at baseline was 63 years (standard deviation: 6.1) and 70% of the participants were women (Table 1) The incidence of dementia, per 1000 person-years, was the lowest in HeSSup (0.7) and the highest in SNAC-K (16.0). Study-specific numbers of participants and dementia cases, by categories of activity participation and duration of follow-up, are provided in Appendix 2.

**Table 1:** Study and participant characteristics

**Study characteristics**	**Health and Social Support study**	**Health 2000**	**Mini-Finland** **follow-up study**	**Swedish National Study on Aging and Care in Kungsholmen**	**Finnish Public Sector study**
Baseline year	1998	2000–2001	2000–2001	2001–2004	2013
End of follow-up	2012	2019	2011	2017	2016
Mean (SD) follow-up, years	14.4 (2.2)	13.6 (6.2)	8.8 (2.5)	8.7 (3.8)	3.1 (0.3)
**Sociodemographic characteristics**					
No. of participants	6073	3793	1057	2740	19,600
*N* (%) women	3312 (54.5)	2169 (57.2)	617 (58.4)	1720 (62.8)	15,741 (80.3)
Mean (SD) age at baseline (years)	52 (1)	65 (11)	64 (9)	74 (11)	64 (6)
Education					
Basic (≤9 years)	2287 (37.7)	2229 (58.8)	507 (48.0)	436 (15.9)	2793 (14.3)
Intermediate (10–12 years)	2862 (47.1)	880 (23.2)	242 (22.9)	1365 (49.8)	6257 (31.9)
High (≥13 years)	924 (15.2)	684 (18.0)	308 (29.1)	939 (34.3)	10,550 (53.8)
**Stimulating leisure-time activities**					
*Activity in specific domains*					
*N* (%) in the highest 4th of participation frequency, by domain					
Mental activity (e.g. reading)	1239 (21.0)	626 (17.6)	364 (37.9)	685 (25.0)	5321 (27.6)
Social activity (e.g. clubs or societies)	1766 (29.7)	713 (20.1)	208 (21.7)	279 (10.2)	5791 (29.9)
Outdoor activity (e.g. gardening)	2454 (41.5)	1476 (42.4)	376 (39.3)	455 (16.6)	11,427 (59.4)
Consumptive activity (e.g. cinema)	1543 (26.0)	854 (24.2)	254 (26.5)	519 (18.9)	2530 (13.1)
Physical activity	1497 (24.8)	1133 (30.8)	374 (35.5)	571 (20.8)	4713 (24.3)
*Variety of activity across domains*					
*N* (%) in the highest 4th of participation frequency, by number of domains					
0	1525 (25.1)	1286 (33.9)	298 (28.2)	1391 (50.8)	4387 (22.4)
1	2007 (33.1)	1059 (27.9)	295 (27.9)	6332 (23.1)	6111 (31.2)
2	1490 (24.5)	847 (22.3)	223 (21.1)	397 (14.5)	5043 (25.7)
≥3	1051 (17.3)	601 (15.8)	241 (22.8)	20 (11.7)	4059 (20.7)
*Frequency of activity across domains*					
*N* (%) by the highest participation frequency					
1	62 (1.0)	257 (6.8)	45 (4.3)	303 (11.1)	281 (1.4)
2	1463 (24.1)	1029 (27.1)	253 (23.9)	1088 (50.8)	4106 (21.0)
≥3	4548 (74.9)	2507 (66.1)	759 (71.8)	1349 (49.2)	15,213 (77.6)
**Covariates**					
*N* (%) with depressive symptoms	1433 (23.8)	1183 (35.9)	311 (33.0)	305 (11.5)	4120 (21.2)
*N* (%) with mobility difficulties	–	561 (17.1)	96 (10.2)	665 (24.7)	2172 (11.3)
*N* (%) with sensory (vision and/or hearing) difficulties	–	1725 (52.4)	309 (32.8)	325 (11.9)	4899 (25.1)
*N* (%) with high-risk alleles of APOE gene	–	998 (30.3)	–	736 (28.8)	–
**Dementia**					
*N* (%) dementia during follow-up	62 (1.0)	751 (19.8)	101 (9.6)	378 (13.8)	116 (0.6)
Incidence per 1000 person-years	0.7	14.6	10.9	16.0	1.9

**Figure 2:**
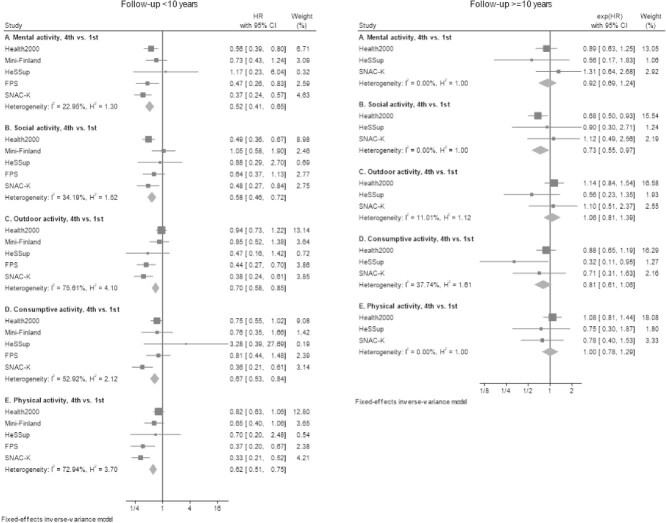
Fixed effects meta-analyses of the association of domain-specific activity with the risk of dementia, by follow-up period. Note to Figure 2: HRs are adjusted for age, sex and education. HRs compare the 4th (most active) to the 1st (least active) quartile of activity participation in each activity domain. Study-specific numbers of participants and dementia cases are presented in appendix, p. 12–13.

Associations of active participation (4th vs. 1st quartile) in mental, social, physical, outdoor and consumptive activities with incident dementia, summarised across the studies by follow-up, are shown in Figure 2. Active participation in all activity domains was associated with a reduced risk of dementia during the first decade of follow-up, with the estimates pointing to 0.52- to 0.70-fold reductions in risk (mental and outdoor activity, respectively). During the second decade of follow-up, the associations of active participation in mental, social, consumptive and physical activity attenuated to the null. The study-specific estimates during the first 10 years of follow-up were heterogenous, particularly for outdoor, physical and consumptive activities, whereas the heterogeneity for all estimates reduced as the follow-up extended to ≥10 years.

**Figure 3:**
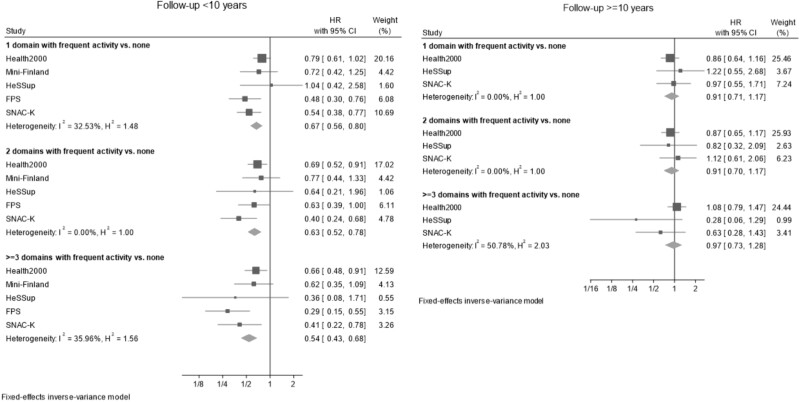
Fixed effects meta-analyses of the association of variety of activities across domains with the risk of dementia, by follow-up period. Note to Figure 3: HRs are adjusted for age, sex and education. HRs compare participants who were in the most active participation quartile (4th) in 1, 2 or ≥3 domains to those who were not in the most active participation quartile in any domain. Study-specific numbers of participants and dementia cases are presented in appendix, p. 14.

The associations of the variety of activities (number of domains in which a person was frequently active) with incident dementia are shown, by follow-up period, in Figure 3. During the first decade of follow-up, the hazard ratio (HR) for dementia comparing individuals who were active in ≥3 domains vs. no domain was 0.54 (95% CI: 0.43 to 0.68). Active participation in two domains vs. none was associated with 0.63-fold risk (95% CI: 0.52 to 0.78) and active participation in one domain vs. none was associated with 0.67-fold risk (95% CI: 0.56 to 0.80). During the second decade of follow-up, the corresponding pooled HRs varied between 0.91 and 0.97, with all 95% confidence intervals (CIs) crossing the null. Heterogeneity among the estimated associations of frequent activity in one or two domains with incident dementia reduced with the extending follow-up, whereas the estimates for an association of activity in ≥3 domains with dementia risk became more heterogeneous in the second follow-up decade.

**Figure 4:**
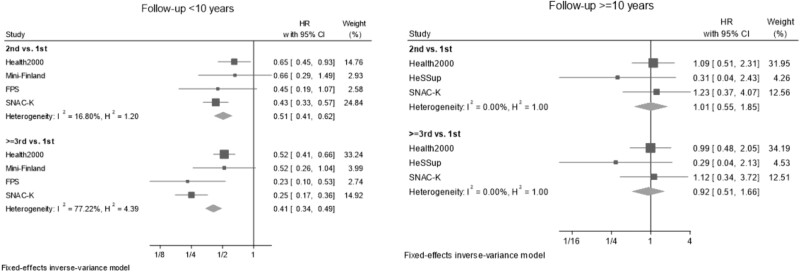
Fixed effects meta-analyses of the association of frequency of activity across domains with the risk of dementia, by follow-up period. Note to Figure 4: HRs are adjusted for age, sex and education. HRs compare individuals with ≥3rd or 2nd as their highest quartile of participation frequency across the domains, to those with the 1st as their highest quartile. Study-specific numbers of participants and dementia cases are presented in appendix, p. 15.

Associations of the overall frequency of activity (the highest activity quartile across the domains) are presented in Figure 4. HRs comparing individuals with ≥3rd as the most active quartile to those with 1st as the most active quartile showed a 0.41-fold risk of dementia (95% CI: 0.34 to 0.49). Estimates comparing individuals with 2nd as the highest quartile to those with 1st as their highest quartile were similar (HR: 0.51, 95% CI: 0.41 to 0.62). The summary estimates indicated no association of the overall activity frequency with dementia during the second decade of follow-up. During the first decade of follow-up, the magnitude of the study-specific estimates varied, but most indicated a protective association; during the second decade of follow-up, the study-specific estimates were heterogeneous in both direction and magnitude, with all 95% CIs crossing the null.

Findings from the sensitivity analyses using a random effects meta-analysis model, restricted to participants aged ≥60 years at baseline, with additional adjustment for baseline depression, mobility difficulties and sensory difficulties, stratified by genetically elevated risk of dementia and with alternative grouping of activities were broadly similar to our main findings (Appendix 2). Findings from additional sensitivity analyses, examining the risk of dementia during the first decade of follow-up only in studies with a follow-up extending to the second decade, were also similar to those of our main findings, suggesting that the characteristics of the studies with shorter and longer follow-up were unlikely to explain the differences in the associations of stimulating activities and the risk of dementia between the two follow-up periods (Appendix 2).

## Discussion

Our findings from five Nordic cohort studies suggest that active participation in mental, social, outdoor, consumptive and physical domains of activity, as well as a larger variety and frequency of activity were associated with reduced risks of dementia in <10 years’ but not in ≥10 years’ follow-up, independently of age, sex and achieved education. Overall, our observations are in line with those of previous studies of leisure-time activity and the long-term dementia risk among older adults, which suggest that the reduced risks of dementia among active individuals during the initial years of follow-up attenuate or are reversed with longer follow-up [9, 11–15]. In a large, prospective study of UK women, non-participation in adult education, arts, crafts, music or voluntary work was strongly associated with an increased risk of dementia in 0–4 years’ follow-up, less strongly in 5–9 years’ follow-up and not at all in ≥10 years’ follow-up [13]. Our analyses of similar follow-up periods had similar findings, but the limited numbers of incident dementia cases in these follow-up categories hampered our ability to reliably estimate the associations during the shorter follow-up intervals. One potential explanation for these findings is that to meaningfully contribute to preserving cognitive function, stimulating activity needs to be longer-term, more intensive or more engaging than many leisure-time pursuits. Indeed, in a multi-cohort investigation including HeSSup, the Finnish Public Sector study and five other prospective studies, the researchers found that cognitive stimulation at work was linked to a reduced short- and long-term risks of subsequent dementia, independently of socioeconomic characteristics and established clinical risk factors [25].

The heterogeneity among the study-specific estimates may relate to differences between study populations and settings. The prevalence and incidence of dementia were the lowest in HeSSup, the study with the youngest participants and the highest in SNAC-K, in which the participants were, on average, the oldest. Health 2000, Mini-Finland, HeSSup and FPS included participants from both urban and rural areas, whereas SNAC-K participants lived in an urban area of Stockholm, and the access to leisure-time activities likely varies across residential locations. The findings from the sensitivity analyses with additional adjustment for characteristics of the participants’ municipality of residence (urban, semi-urban or rural) did not markedly differ from our main findings, but it is possible that the municipality-level data were not sufficiently representative of the availability of or access to leisure-time activity options. The types and frequency of activities are also likely to be influenced by individual socioeconomic circumstances. We adjusted all the estimates for achieved education, which is an important indicator of socioeconomic position, as well as associated with the risk of dementia [16, 17]. It is also possible that the participants in FPS (occupational cohort participants who had left employment at the participating institutions) and Mini-Finland Follow-up Study (older individuals who were willing and able to take part in a follow-up of a previous investigation) were healthier and more active in their leisure-time than the general population of old adults. This may have biased the prevalence and incidence estimates in these studies downwards, but evidence suggests that exposure-disease associations in these types of observational cohort studies are often representative of the same associations in the wider target population [26, 27].

Our findings may reflect two, potentially co-existing processes. The first one is reverse causality, whereby individuals with prodromal dementia reduce their leisure-time activity. The second one is the dilution of the effect of activity over the follow-up period, potentially exacerpated by a reducing number of participants and dementia cases over an extended follow-up. Repeated assessments of leisure-time activity participation over a long follow-up would help to examine the dilution hypothesis further.

The exact mechanism linking cognitive and social stimulation to dementia is unclear. One possibility is that cognitively, socially or physically stimulating activity impacts on dementia risk via its influence on other cardiometabolic or lifestyle-related risk factors. For example, diabetes, smoking, excessive alcohol consumption and physical inactivity, which have been identified as important preventive targets in dementia [28], could be influenced by regular engagement in stimulating leisure-time activities, e.g. by reducing sedentary time or increasing the amount of regular social contact. The findings from a multi-cohort study of work-related cognitive stimulation and the risk of dementia also pointed towards a biological mechanism, showing that cognitive stimulation at work was associated with lower levels of plasma proteins that might inhibit axonogenesis and synaptogenesis and increase dementia risk [25]. Previous research suggests that cumulative exposure to factors that enhance cognitive reserve across the lifespan is associated with reduced risk of dementia in late life [29]. Our observations of stimulating leisure-time activities being associated with a reduced risk of dementia in short-term but not in the long term suggest that the beneficial effects of stimulating activities can wear out over time. Taken together, these findings suggest that whilst engaging in stimulating activities have health benefits at any age, activity in later life is important.

### Strengths and limitations

A strength of our investigation is that we utilised data from five independent, prospective studies with reasonable participation rates and low attrition. In SNAC-K, dementia diagnoses were made by clinicians as a part of the follow-up examinations, and in the Finnish studies, dementia was ascertained from specialised healthcare, prescription medications and death registers, which have good coverage and accuracy for identifying chronic diseases [30, 31]. However, our use of specialised healthcare data means that some cases of mild or early stages of dementia, managed in primary care, may have been misclassified as free of this disease [32]. Also, these disease records do to provide an exact timing of the disease onset as dementia has a long prodromal period and there is variation in health service provision and patients’ and their families care seeking behaviour. Unfortunately, we did not have access to good quality primary care data for our study period and were unable to explore these issues further.

Our findings were similar across activity domains as well as the variety and frequency of participation across the domains, and robust to adjustment for baseline age, sex, achieved education, depression, mobility difficulties and sensory difficulties. It is, of course, possible that our observations have been influenced by residual confounding from unknown or unmeasured confounders (e.g. head injuries or circulating plasma proteins or lipids).

The accuracy of older adults’ self-reporting of average or typical leisure-time activity varies [33] and it is possible that error in the self-reported activity has diluted our association estimates. However, as randomising individuals to cognitively stimulating activities or objectively measuring activity participation are often unfeasible, the evidence on the link between cognitive stimulation and the risk of dementia must mainly come from observational studies. Overall, individual studies in our investigation had modest numbers of dementia cases, which reduced the analytical power. To overcome this limitation and to examine variation in the observed associations, we pooled the study-specific estimates in meta-analyses, which provided evidence for generally consistent patterns of associations across the studies. As all the studies were conducted in ethnically homogeneous, predominantly European populations, the generalisability of our findings outside of the Nordic context or high-income settings is uncertain. Investigating the associations of participating in stimulating leisure-time activities with the risk of dementia in middle- and low-income settings and diverse environmental and cultural contexts would be of interest.

## Conclusions

Our findings suggest that active participation in stimulating leisure-time activities is associated with a reduced risk of dementia in a follow-up of up to 10 years, but not beyond that. These observations may reflect a dilution of the association over time or reverse causality.

## Supplementary Material

aa-23-1729-File002_afae141
