# Supplementary material for: Stimulating leisure-time activities and the risk of dementia: a multi-cohort study
Source: Age Ageing. 2024 Jul 13;53(7):afae141. doi: 10.1093/ageing/afae141 (PMC11246193; doi:10.1093/ageing/afae141)
Supplement: aa-23-1729-File002_afae141 [file aa-23-1729-file002_afae141.pdf]

Appendices

Stimulating leisure-time activities and the risk of dementia: a multi-cohort study

Katriina Heikkilä, Jaana Pentti, Serhiy Dekhtyar, Jenni Ervasti, Laura Fratiglioni, Tommi Härkänen, Mika Kivimäki, Seppo Koskinen, Tiia Ngandu, Säde Stenlund, Sakari Suominen, Jussi Vahtera, Suvi Rovio, Sari Stenholm

Contents

Contents	Page
Appendix 1. Extended methods	1-9
Appendix 2. Extended results	10-27
Appendix 3. Extended references	29

Appendix 1. Extended methods

Study population

We used individual-level data from five prospective cohort studies: Health and Social Support (HeSSup), Health 2000, Mini-Finland Follow-up Study and Finnish Public Sector study (FPS) from Finland and Swedish National Study on Aging and Care in Kungsholmen (SNAC-K) from Sweden (figure 1). HeSSup is a prospective cohort study based on a stratified random sample of the Finnish population in four age groups (20–24, 30–34, 40–44, and 50–54 years), identified from the nationwide population register in 1998.[1] Data were collected by self-completed questionnaires and the participants' records were linked to nationwide healthcare registers and population data for information on disease outcomes and death. Health 2000 Survey is based on a two-stage, stratified cluster sample of men and women aged 30 year or older who were identified from the nationwide population register and invited to take part in the health survey and examination in 2000. [2 3] In addition, individuals who had participated in a previous study, Mini-Finland Health Survey, in 1978-1980, and remained alive and living in one of the seven large cities in Finland (Helsinki, Turku, Salo, Lahti, Tampere, Kuopio or Oulu) in 2000 were invited to participate.[3 4] The data collection included self-completed questionnaires and a health examination, including blood sampling, and record linkage to nationwide registers for health outcomes and death.[5] SNAC-K is a prospective cohort study based on a stratified, random sample of individuals who lived on Kungsholmen in central Stockholm. After a baseline assessment in 2001-2004, participants aged 60, 66 and 72 years were invited to follow-up examinations every six years and those aged ≥78 years were re-examined every three years. [6] FPS is a prospective cohort study of all public sector personnel of 10 towns and 21 hospitals, recruited from the employers' records in 1997 and followed-up by record-linkage to nationwide registers for health outcomes and death.[7] We used data from a sub-sample of individuals who had left employment in the participating organisations by 2013. In all five cohort studies our analyses were based on data from participants who were aged ≥ 50 years at analytical baseline when stimulating leisure-time activities were ascertained and who had available data on at least one stimulating leisure-time activity, age, sex and education and no record of dementia during or before the baseline year (figure 1).

Ethical approvals

HeSSup was approved by the joint Ethical Committee of the University of Turku and the Turku University Central Hospital. Health 2000, including Mini-Finland Follow-up Study, was approved by Helsinki and Uusimaa hospital district Ethics Committee for Epidemiology and Public Health. Health 2000 and Mini-Finland Follow-up Study genotyping was based on study specific consent and

the transfer of the samples and data to the Finnish Institute for Health and Welfare Biobank was approved by Valvira, the National Supervisory Authority for Welfare and Health. FPS was approved by Helsinki and Uusimaa Hospital District ethics committee (HUS/1210/2016). SNAC-K was approved by Karolinska Institutet Ethics Committee and the Regional Ethical Review Board in Stockholm. The participants (or a proxy in SNAC-K) in all studies provided written, informed consent.

Stimulating leisure-time activities

Cognitively, socially or physically stimulating leisure-time activities in all studies were ascertained from self-report questionnaires at baseline: 1998 in HeSSup, 2000-2001 in Health 2000 and Mini-Finland Follow-up Study, 2001-2004 in SNAC-K and 2013 in FPS. Participants responded to questions on the type and frequency of various leisure-time activities. Activities were divided into five domains: mental, social, outdoor, consumptive and physical activity (appendix, supplementary table 1). Mental activity included studying, reading books, newspapers or magazines, arts and crafts, playing an instrument, photography, painting, collecting items, cooking, baking, shopping and taking care of finances at the bank. Social activity included participation in clubs or societies, visiting with family, friends or neighbours and chatting on the telephone. Outdoor activity comprised hunting, fishing and gardening. Consumptive activity included going to the cinema, theatre, concerts, exhibitions, religious events, cafes, bars, restaurants or night clubs, ie. activities involving consuming material or non-material creations of others. Physical activity included walking, jogging, running, exercise and sports. Study participants indicated the frequency of taking part in each activity on Likert scales, with response options ranging from daily or most days to never. In HeSSup and FPS, the responses to the questions on physical activity were converted into metabolic equivalent of task (MET)-hours per week. The mean response for each activity domain (mental, social, outdoor, consumptive and physical) was calculated for each participant; the mean scores across participants were divided into quartiles and participants whose score was in the highest quartile (4th) were defined as active participants in the relevant activity domain. In addition, we calculated two overall measures of activity: variety and overall frequency. The variety of activity was operationalised as the number of activity domains in which a participant's mean score was in the highest quartile of participation; the overall frequency of activity was operationalised as the participant's highest activity participation quartile across the five domains, regardless of how many domains were involved

Dementia

Dementia was ascertained from diagnostic codes and dates recorded in the nationwide hospital care register (maintained by the Finnish Institute for Health and Welfare), medication purchase and special reimbursement register (maintained by the Social Insurance Institution) and causes of death data (maintained by Statistics Finland) in the Finnish studies. In Health 2000 and Mini-Finland, information on in-patient care data were available from 1st January 1969, medication reimbursement data from 1st January 1964, medications purchase data from 1st January 1995 and out-patient care data from 1st January 1998 onwards, with the register follow-up extending to 31st December 2011 in Mini-Finland, 31st December 2019 in Health 2000.[8] In HeSSup, all healthcare register data were available from 1st January 1998 to 31st December 2012 and in FPS from 1st January1980 to 31st December 2016. In the Finnish studies, participants were defined as having dementia if they had hospital in-patient or out-patient records or death register records containing International Classification of Diseases (ICD) versions 8 code 290, version 9 codes 290, 3310 and 4378A or version 10 codes F00-F03 and G30; medication purchase records with Anatomical Therapeutic Chemical (ATC) code N06D (anti-dementia medications: donepezil, rivastigmine, galantamine and memantine) or medication reimbursement code 307 (up to 2015: donepezil, rivastigmine, galantamine and memantine; from 2016 to 2018: rivastigmine). In SNAC-K, participants were followed up by regular health examinations up to 31st December 2017, with dementia ascertained from structured interviews, clinical examination and cognitive testing. Dementia was ascertained clinically in accordance with Diagnostic and Statistical Manual of Mental Disorders version 4 (DSM-IV) criteria. A three-step procedure was employed, whereby two physicians working independently made a preliminary diagnosis and, where necessary, a third opinion was obtained from a senior neurologist. Medical records and death certificates of participants who died during the follow-up period were also collected and reviewed by physicians to determine whether the participants died with dementia.[9]

Covariates

Age and sex were ascertained from Statistics Finland population data in Mini-Finland Follow-up Study, Health 2000 and HeSSup. In FPS, age and sex were ascertained from the employer's records. Education was ascertained from baseline self-reporting questionnaire in HeSSup, Health 2000 and Mini-Finland Follow-up Study and from Statistics Finland data in FPS. In SNAC-K, age, sex and education were self-reported. Education was categorised into basic (≤9 years), intermediate (10-12 years) or high (≥ 13 years). Depressive symptoms were ascertained from self-reported questionnaire responses to Beck Depression Inventory [10] in HeSSup, Health 2000 and Mini-Finland (with a score ≥10 points indicating depressive symptoms), Montgomery-Åsberg depression rating scale[11] in

SNAC-K (with a score ≥7 points indicating depressive symptoms) and questions on recently experienced depression, hopelessness or lack of interest in FPS. Mobility and sensory difficulties were ascertained from responses to questions on difficulties in walking 500m[12], seeing or hearing, and analysed as binary variables (any difficulty vs. no difficulty) in all studies. To investigate the impact of genetically increased risk of dementia on our findings, we conducted sensitivity analyses stratified by *APOE* ε4 carrier status [13] in Health 2000 and SNAC-K. In Health 2000 baseline fasting blood samples were genotyped using Illumina CoreExome, OMNIExpress and 610K arrays. Individuals with non-European ancestry or obscure sex were excluded from genotyping. Quality control prior to phasing and imputation excluded variants with missingness >5%, call rate <95%, minor allele count (MAC) <3 (if Zcalled) or MAC <10 (if called using Illumina GenCal), INFO <0.8, minor allele frequency <0.001%, Hardy-Weinberg equilibrium p-value <1*10-10, and heterozygosity exceeding ±4 standard deviations. Quality control was performed simultaneously on all data. Prior to imputation, haplotypes were estimated using SHAPEIT2.[14] Imputation was undertaken with IMPUTE2 [15] using high-coverage, population-specific reference panels of 2690 whole-genome and 5093 whole-exome sequences.[16] In SNAC-K, genomic DNA was extracted from peripheral blood samples collected at baseline. *APOE* ε4 carrier status was determined using a microsequencing method (AffiGen APOE; Sangtec Medical, Stockholm, Sweden) based on a polymerase chain reaction with biotinylated primers.[17]

Statistical analyses

The association of stimulating leisure-time activities with the risk of dementia was examined using Cox proportional hazards regression models in all studies. We examined active participation in specific activity domains, variety of activity and the overall frequency of activity. The proportional hazards assumption was checked visually, by inspecting log-(-log) plots. The timescale in the models was age.[18] The period under observation began on the date of baseline participation or the date when stimulating activities were ascertained and ended on the first of the following: the date of specialised healthcare record of dementia, the date of prescription or purchase of medication for dementia, date of emigration from Finland (in Health 2000 and Mini-Finland; HeSSup and FPS did not include information on emigration dates) or Sweden (SNAC-K), date of death or the end of register follow-up. In SNAC-K, the end of follow-up was the date when the participant was last contacted. In all study-specific analyses using Health 2000 data, Stata's survey commands with baseline sampling weights were used to adjust for the unequal probability of the participants being included in the study. Individuals with a record of dementia during the baseline year were defined as having dementia at or before baseline and exclude from the analyses (figure 1).

To examine the extent to which reverse causality had affected the association estimates, we modelled the association of the activity exposures with dementia during two time periods: <10 years and ≥10 years following activity ascertainment.[19] Preliminary analyses with the follow-up periods split into <5 years, 5-9 years, 10-14 years and ≥15 years were also conducted. However, in the interest of analytical power and the comparability of the findings to those of previous studies, the main analyses were conducted with the follow-up time split into two time periods (<10 years and ≥10 years).

We tested for multiplicative interaction by including an activity*education interaction term in our main models. As the overall number of studies was small, we pooled study-specific estimates in fixed effects meta-analysis.[20 21]

We conducted five sets of sensitivity analyses. We ran the main analyses (1) using Sidik-Jonkman between-study variance estimator, which previous research suggest performs well with sparse data.[20 21]; (2) restricting the study population to people aged ≥ 60 years at baseline; (3) with additional adjustment for baseline depression, sensory difficulties and mobility difficulties in a subset of studies with these data available; (4) stratified by genetic risk (i.e. separately for individuals with any number of *APOE* ε4 alleles and those with no high-risk alleles) in Health 2000 and SNAC-K; and (5) using an alternative, previously used grouping of activities in SNAC-K.[22 23] Using this approach, leisure-time activities were categorised into mental activity (reading books, playing chess/cards, playing a musical instrument, listening to music, using the internet or playing computer games, and painting/drawing/working with clay), social activity (attending sports events, cinema, theatre, concerts, museums, art exhibitions, restaurants, bar, cafés, bingo, dancing or church services, travelling, volunteering and attending study circles or courses) and physical activity (walking, jogging, bicycling, going to the gym, playing golf or other sports, gardening, strolling through the woods and countryside, picking mushrooms or berries, going hunting or fishing, home repair and car/other mechanical repairs) (supplementary table 1).

All meta-analyses and the study-specific analyses in Health 2000, Mini-Finland Follow-up Study and SNAC-K were conducted using Stata SE 17 (Stata Corporation, College Station, Texas, USA). The study-specific analyses in FPS and HeSSup were conducted using SAS 9.4 (/SAS Institute, Cary, North Carolina, USA).

Supplementary table 1. Ascertainment of stimulating activities from self-reporting questionnaires

Stimulating leisure-time activity	Health and Social Support Study (HeSSup)	Health 2000 and Mini-Finland	Swedish National study on Aging and Care in Kungsholmen (SNAC-K)	Finnish Public Sector Study (FPS)
**Mental activity**				
Question	*How often have you spent your free time doing these activities in the past years?*	*How often do you typically undertake these activities?*	*Have you participated in any of the following entertainment or cultural activities in the past 12 months?*	*How often have you spent your free time doing these activities in the past years?*
	Studying in free time (e.g. community college, by myself)	Studying	Reading books	Studying
	Writing, singing, playing an instrument, drawing, painting, dancing, photography or other creative hobby	Reading books, listening to records or tapes	Reading newspapers	Crafts, handiwork, playing an instrument, singing, photography, painting, collecting items
	Reading as a hobby	Reading newspapers or magazines	Reading magazines/journals	
		Arts and crafts, playing an instrument, singing, photography, painting, collecting items	Playing chess/card games	
		Cooking or baking	Using the internet/playing computer games	
		Shopping or visiting the bank	Playing an instrument	
			Attending a study group or course	
			Knitting, weaving, sewing	
			Painting, drawing, working with clay, pottery	
			Home repairs	
			Car/mechanical repairs	
Response options	*Weekly or more often (4)*<br>*1-3 times a month (3)*<br>*Once or a few times a year (2)*<br>*Never (1)*	*Daily or most days (5)*<br>*Once or twice a week (4)*<br>*Once or twice a month (3)*<br>*Once or a few times a year (2)*<br>*More rarely or never (1)*	*Weekly*<br>*A few times per month*<br>*Less frequently*<br>*Never*	*Every day or most days of the week (5)*<br>*Once or twice a week (4)*<br>*Once or twice a month (3)*<br>*Once or twice a year (2)*<br>*More rarely or never (1)*
Categorisation (Q: quartile)	Q1: <=1.7<br>Q4: >=3.3	Health 2000<br>Q1: >=3<br>Q4: >=4.17<br><br>Mini-Finland<br>Q1:<=2.3<br>Q4: >=5	Q1: <=0.45<br>Q4: >=1.09	Q1: <=1.5<br>Q4: >=3.5
**Social activity**				
Question	*How often have you spent your free time doing these activities in the past years?*	*How often do you typically undertake these activities?*	*Have you participated in any of the following entertainment or cultural activities in the past 12*	*How often have you spent your free time doing these activities in the past years?*

			months?	
	Visiting with family or friends	Participating in clubs or societies	Participating in clubs or societies	Clubs or societies
	Participating in clubs or societies, voluntary work, other societal participation	Visiting friends or neighbours	Voluntary work	Visiting family, friends or neighbours
		Having friends or neighbours visit		Having family, friends or neighbours visit at home
		Talking on the telephone		Talking on the telephone
Response options	*Weekly or more often (4)*<br>*1-3 times a month (3)*<br>*Once or a few times a year (2)*<br>*Never (1)*	*Daily or most days (5)*<br>*Once or twice a week (4)*<br>*Once or twice a month (3)*<br>*Once or a few times a year (2)*<br>*More rarely or never (1)*	*Weekly*<br>*A few times per month*<br>*Less frequently*<br>*Never*	*Every day or most days of the week (5)*<br>*Once or twice a week (4)*<br>*Once or twice a month (3)*<br>*Once or twice a year (2)*<br>*More rarely or never (1)*
Categorisation	Q1: <=1.5<br>Q4: >=3	Health 2000<br>Q1:<=2.75<br>Q4: >=3.67<br><br>Mini-Finland<br>Q1: <=2.25<br>Q4:>=3.5	Q1: <=1.5<br>Q4: >=3	Q1: <=2.75<br>Q4: >=3.75
**Outdoor activity**				
Question	*How often have you spent your free time doing these activities in the past years?*	*How often do you typically undertake these activities?*	*Have you participated in any of the following entertainment or cultural activities in the past 12 months?*	*How often have you spent your free time doing these activities in the past years?*
	Hunting, fishing or boating	Exercise, hunting, fishing, gardening or other outdoor activity	Gardening	Exercise, hunting, fishing, gardening or other outdoor activities
	Physical activity, sports or exercise		Hiking in woods, berry or mushroom picking	
			Hunting or fishing	
Response options	*Weekly or more often (4)*<br>*1-3 times a month (3)*<br>*Once or a few times a year (2)*<br>*Never (1)*	*Daily or most days (5)*<br>*Once or twice a week (4)*<br>*Once or twice a month (3)*<br>*Once or a few times a year (2)*<br>*More rarely or never (1)*	*Weekly*<br>*A few times per month*<br>*Less frequently*<br>*Never*	*Every day or most days of the week (5)*<br>*Once or twice a week (4)*<br>*Once or twice a month (3)*<br>*Once or twice a year (2)*<br>*More rarely or never (1)*
Categorisation (Q: quartile)	Q1: <=2<br>Q4: >=3	Health 2000<br>Q1: <=3<br>Q4: >=5<br><br>Mini-Finland<br>Q1: <=1<br>Q4: >=5	Q1: <=0.33<br>Q4: >=1.67	Q1: <=3<br>Q4: =5
**Consumptive activity**				

Question	*How often have you spent your free time doing these activities in the past years?*	*How often do you typically undertake these activities?*	*Have you participated in any of the following entertainment or cultural activities in the past 12 months?*	*How often have you spent your free time doing these activities in the past years?*
	Participating in spiritual or religious events	Going to the cinema, theatre, concerts or sporting events	Going to cinema, theatre or concerts	Attending church or other religious events
	Going to cultural or entertainment events, e.g. cinema, concerts, theatre, art exhibitions or similar	Going to religious events	Going to sporting events	Going to theatre, concerts, art exhibitions, sporting events or similar
	Going to restaurants, discos, pubs or dancing	Visiting restaurants, bars or night clubs for recreation	Going to museums or art exhibitions	
			Going to restaurants, pubs or cafes	
			Bingo	
			Dancing	
			Attending church or revival meetings	
Response options	*Weekly or more often (4)*<br>*1-3 times a month (3)*<br>*Once or a few times a year (2)*<br>*Never (1)*	*Daily or most days (5)*<br>*Once or twice a week (4)*<br>*Once or twice a month (3)*<br>*Once or a few times a year (2)*<br>*More rarely or never (1)*	*Weekly*<br>*A few times per month*<br>*Less frequently*<br>*Never*	*Every day or most days of the week (5)*<br>*Once or twice a week (4)*<br>*Once or twice a month (3)*<br>*Once or twice a year (2)*<br>*More rarely or never (1)*
Categorisation (Q: quartile)	Q1: <=1.5<br>Q4: >=2.3	Health 2000<br>Q1: <=1.3<br>Q4>=2.3<br>Mini-Finland<br>Q1: <=1.5<br>Q4: >=3	Q1: <=0.43<br>Q4: >=1.28	Q1: <=1.5<br>Q4: >=3
**Physical activity**				
Question	*How much physical activity have you done in your free time or whilst commuting during the past year, on average?*	*How often do you exercise for at least 30 minutes at a time so that you sweat and get out of breath?*	*Do you regularly engage in* ***light exercise*** *(e.g. walking along roads or in parks, walking in the woods, short bicycle rides, light aerobics, golf)?*	*How much physical activity have you done in your free time or whilst commuting during the past year, on average?*
	Walking or equivalent			Walking or equivalent
	Brisk walking or equivalent			Brisk walking or equivalent
	Light running, jogging or equivalent		*Do you regularly engage in m**oderate to intense** exercise, now or previously (e.g. jogging, long power walks, heavy-duty gardening, long bicycle rides, high-intensity aerobics, long distance ice skating, swimming, ball sports (not golf) or other similar activity)?*	Light running, jogging or equivalent
	Brisk running or equivalent			Brisk running or equivalent

Response options	*2-3 hours per week*<br>*about an hour per week*<br>*Less than half an hour per week*<br>*None at all*<br><br>Responses converted into metabolic equivalent of task (MET) and weekly physical activity expressed as MET-hours per week.	*Daily (6)*<br>*4-6 times per week (5)*<br>*2-3 times per week (4)*<br>*Once per week (3)*<br>*2-3 times per month (2)*<br>*A few times per year or more rarely (1)*		*Weekly*<br>*A few times per month*<br>*Less frequently*<br>*Never*	*2-3 hours per week*<br>*about an hour per week*<br>*Less than half an hour per week*<br>*None at all*<br><br>Responses converted into metabolic equivalent of task (MET) and weekly physical activity expressed as MET-hours per week.
Categorisation	Q1: met<=10<br>Q4: met>35.5	Health 2000<br>Q1: <=2<br>Q2: >=5	Mini-Finland<br>Q1: <=2<br>Q4: >=5	Q1: <=1<br>Q4: >=3	Q1: met<=9.9<br>Q4: met>32

Appendix 2. Extended results

Supplementary table 2. Study-specific associations of activities with the risk of dementia in <5, 5-9, 10-14 and ≥15 years' follow-up

			Follow-up <5 years						Follow-up 5-9 years						Follow-up 10-14 years						Follow-up >=15 years					
Study	Activity	Quartile	N participants	N cases	% cases	Adjusted HR	95% CI		N participants	N cases	% cases	Adjusted HR	95% CI		N participants	N cases	% cases	Adjusted HR	95% CI		N participants	N cases	% cases	Adjusted HR	95% CI	
**Health2000**	Mental	1st	1042	87	8.4	1			746	74	9.9	1			539	55	10.2	1			395	32	8.1	1		
		2nd-3rd	1875	58	3.1	0.44	0.3	0.64	1676	99	5.9	0.78	0.56	1.09	1416	96	6.8	0.62	0.43	0.87	1187	79	6.7	0.85	0.54	1.32
		4th	624	12	1.9	0.3	0.16	0.57	573	31	5.4	0.78	0.5	1.24	500	41	8.2	0.73	0.48	1.12	420	33	7.9	1.13	0.65	1.95
	Social	1st	1062	66	6.2	1			831	71	8.5	1			651	55	8.5	1			521	47	9.0	1		
		2nd-3rd	1759	69	3.9	0.68	0.47	0.98	1 530	97	6.3	0.74	0.55	1.01	1282	92	7.2	0.77	0.55	1.08	1066	69	6.5	0.66	0.46	0.96
		4th	713	23	3.2	0.42	0.26	0.7	632	37	5.9	0.51	0.34	0.78	520	45	8.7	0.75	0.5	1.11	415	29	7.0	0.62	0.38	1.01
	Outdoor	1st	915	70	7.7	1			639	55	8.6	1			449	33	7.4	1			358	23	6.4	1		
		2nd-3rd	1083	36	3.3	0.79	0.51	1.23	981	45	4.5	0.98	0.65	1.50	850	70	8.2	1.47	0.97	2.24	712	37	5.2	0.89	0.52	1.52
		4th	1473	42	2.9	0.62	0.4	0.93	1333	97	7.3	1.169	0.84	1.69	1131	89	7.9	1.11	0.74	1.65	916	80	8.7	1.23	0.76	1.98
	Consumptive	1st	1010	80	7.9	1			742	73	9.8	1			528	47	8.9	1			408	48	11.8	1		
		2nd-3rd	1658	61	3.7	0.69	0.48	0.99	1452	86	5.9	0.89	0.64	1.24	1235	89	7.2	1.01	0.71	1.43	1022	70	6.9	0.7	0.48	1.02
		4th	853	15	1.8	0.42	0.24	0.75	790	45	5.7	0.94	0.34	1.39	686	56	8.2	1.14	0.77	1.69	571	27	4.7	0.53	0.32	0.86
	Physical	1st	1009	96	9.5	1			726	57	7.9	1			549	51	9.3	1			436	26	6.0	1		
		2nd-3rd	1 524	40	2.6	0.48	0.31	0.72	1 376	73	5.3	1.14	0.79	1.64	1187	72	9.5	0.77	0.54	1.11	1000	53	5.3	0.86	0.53	1.38
		4th	1 131	38	3.4	0.46	0.31	0.69	987	79	10.8	1.27	0.89	1.82	794	75	9.5	0.94	0.66	1.35	630	66	10.5	1.37	0.86	2.18
**Mini-Finland**	Mental	1st	175	15	8.6	1			141	10	7.1	1														
		2nd-3rd	413	14	3.4	0.77	0.36	1.64	379	15	4.0	0.87	0.38	1.97												
		4th	361	14	3.9	0.57	0.27	1.23	325	22	6.8	0.98	0.46	2.11												
	Social	1st	303	13	4.3	1			269	10	3.7	1														
		2nd-3rd	440	15	3.4	0.8	0.37	1.69	393	28	7.1	1.60	0.77	3.32												
		4th	205	14	6.8	1.16	0.53	2.53	183	9	4.9	0.87	0.35	2.19												
	Outdoor	1st	242	18	7.4	1			199	11	5.5	1														
		2nd-3rd	332	8	2.4	0.63	0.27	1.47	302	14	4.6	1.08	0.48	2.41												
		4th	372	16	4.3	0.7	0.35	1.40	342	22	6.4	1.07	0.52	2.23												
	Consumptive	1st	105	7	6.7	1			86	3	3.5	1														
		2nd-3rd	589	28	4.9	0.90	0.39	2.10	524	31	5.9	1.74	0.53	5.34												
		4th	253	6	2.4	0.41	0.13	1.25	235	13	5.5	1.5	0.42	5.37												
	Physical	1st	230	21	9.1	1			173	12	6.9	1														
		2nd-3rd	442	13	2.9	0.50	0.24	1.03	405	16	4.0	0.67	0.31	1.45												
		4th	367	13	3.5	0.46	0.22	0.97	339	26	7.7	0.86	0.42	1.75												
**HeSSup**	Mental	1st	1233	2	0.2	1			1208	1	0.1	1			1173	7	0.6	1								
		2nd-3rd	3469	4	0.1	0.66	0.12	3.80	3402	14	0.4	5.80	0.75	44.85	3295	23	0.7	0.99	0.42	2.37						
		4th	1253	1	0.1	0.45	0.04	5.54	1235	2	0.2	2.59	0.23	29.63	1203	5	0.4	0.56	0.17	1.84						
	Social	1st	958	0	0.0				934	5	0.5	1			898	5	0.6	1								
		2nd-3rd	3257	5	0.2	NA			3194	7	0.2	0.43	0.13	1.35	3102	22	0.7	1.22	0.46	3.23						
		4th	1781	3	0.2	NA			1755	5	0.3	0.56	0.16	1.95	1709	9	0.5	0.91	0.30	2.71						
	Outdoor	1st	1403	1	0.1	1			1367	6	0.4	1			1310	10	0.8	1								
		2nd-3rd	2088	4	0.2	2.75	0.31	24.82	2054	7	0.3	0.82	0.27	2.45	1997	14	0.7	0.87	0.39	1.98						
		4th	2466	2	0.1	1.00	0.09	11.04	2430	4	0.2	0.38	0.11	1.37	2370	10	0.4	0.56	0.23	1.35						
	Consumptive	1st	745	0	0.0				730	1	0.1	1			696	8	1.2	1								
		2nd-3rd	3667	3	0.1	NA			3596	14	0.4	3.18	0.42	24.39	3498	21	0.6	0.49	0.22	1.12						
		4th	1562	4	0.3	NA			1538	2	0.1	1.16	0.10	13.04	1492	6	0.4	0.32	0.11	0.94						
	Physical	1st	1607	2	0.1	1			1564	4	0.3	1			1504	11	0.7	1								
		2nd-3rd	2967	4	0.1	1.09	0.20	6.04	2913	10	0.3	1.35	0.42	4.34	2834	16	0.6	0.72	0.33	1.55						
		4th	1510	2	0.1	1.04	0.15	7.39	1488	2	0.1	0.53	0.10	2.89	1448	8	0.6	0.75	0.30	1.87						
**FPS**	Mental	1st	3574	32	0.9	1																				
		2nd-3rd	10399	58	0.6	0.66	0.43	1.02																		
		4th	5321	20	0.4	0.47	0.26	0.83																		
	Social	1st	4046	24	0.6	1																				
		2nd-3rd	9523	61	0.6	1.03	0.64	1.65																		
		4th	5791	27	0.5	0.65	0.37	1.13																		
	Outdoor	1st	2351	26	1.1	1																				
		2nd-3rd	5462	29	0.5	0.51	0.30	0.87																		
		4th	11427	54	0.5	0.44	0.27	0.70																		
	Consumptive	1st	5334	37	0.7	1																				
		2nd-3rd	11429	58	0.5	0.70	0.46	1.07																		
		4th	2530	16	0.6	0.81	0.44	1.48																		
	Physical	1st	4698	46	1.0	1																				
		2nd-3rd	9979	52	0.5	0.57	0.38	0.85																		
		4th	4713	14	0.3	0.37	0.20	0.68																		
**SNAC-K**	Mental	1st	754	100	13.3	1			424	64	15.1	1			195	13	6.7	1			2	0	NA			
		2nd-3rd	1301	30	2.3	0.36	0.23	0.55	1124	77	6.9	0.68	0.48	0.97	771	46	6	1.73	0.91	3.28	10	0	NA			
		4th	684	7	1.0	0.28	0.13	0.63	642	20	3.1	0.41	0.24	0.70	508	21	4.1	1.31	0.64	2.69	4	0	NA			
	Social	1st	1019	109	10.7	1			639	77	12.1	1			327	22	6.7	1			4	0	NA			
		2nd-3rd	1442	27	1.2	0.44	0.28	0.69	1288	71	5.5	0.72	0.52	1.02	957	50	5.2	0.54	0.91	2.62	10	0	NA			
		4th	279	1	0.4	0.10	0.01	0.70	263	13	4.9	0.69	0.38	1.26	190	8	4.2	1.12	0.49	2.57	2	0	NA			
	Outdoor	1st	747	103	13.8	1			420	60	14.3	1			193	16	8.3	1			2	0	NA			
		2nd-3rd	1538	32	2.1	0.33	0.21	0.50	1347	83	6.2	0.67	0.47	0.95	943	50	5.3	1.07	0.59	1.95	12	0	NA			
		4th	455	2	0.4	0.10	0.03	0.42	423	18	4.3	0.58	0.33	1.01	338	14	4.1	1.09	0.51	2.35	2	0	NA			
	Consumptive	1st	710	96	13.5	1			396	61	15.4	1			184	16	8.7	1			3	0	NA			
		2nd-3rd	1511	38	2.5	0.37	0.25	0.54	1309	86	6.6	0.59	0.42	0.83	891	54	6.1	1.13	0.63	2.02	11	0	NA			
		4th	519	3	0.6	0.18	0.06	0.59	485	14	2.9	0.43	0.24	0.80	399	10	2.5	0.71	0.31	1.63	2	0	NA			
	Physical	1st	699	87	12.5	1			392	57	14.5	1			195	18	9.2	1			4	0	NA			
		2nd-3rd	1470	44	3.0	0.42	0.29	0.61	1273	87	6.8	0.60	0.43	0.85	854	45	5.3	0.87	0.50	1.51	9	0	NA			
		4th	571	6	1.1	0.28	0.12	0.65	525	17	3.2	0.35	0.20	0.62	425	17	4	0.79	0.40	1.54	3	0	NA			

Note: Quartile refers to the frequency of participation in each activity, divided into quartiles.
Hazard ratios are adjusted for age, sex and education.

Supplementary table 3. Study-specific associations of activities with the risk of dementia in <10 years' follow-up

Study	Activity	Quartile	N participants	N cases	% cases	Unadjusted HR	95% CI		Adjusted HR	95% CI		p for interaction with education
Health2000	Mental	1st	1042	161	15.5	1			1			
		2nd-3rd	1875	157	8.4	0.68	0.54	0.85	0.64	0.5	0.81	
		4th	624	43	6.9	0.62	0.44	0.57	0.56	0.39	0.8	0.9
	Social	1st	1062	137	12.9	1			1			
		2nd-3rd	1759	166	9.4	0.75	0.6	0.95	0.74	0.58	0.93	
		4th	713	60	8.4	0.5	0.37	0.69	0.49	0.36	0.67	0.2
	Outdoor	1st	915	125	13.7	1			1			
		2nd-3rd	1083	81	7.5	0.91	0.68	1.24	0.91	0.68	1.23	
		4th	1473	139	9.4	0.94	0.73	1.22	0.94	0.73	1.22	0.9
	Consumptive	1st	1010	153	15.2	1			1			
		2nd-3rd	1658	147	8.9	0.83	0.65	1.05	0.83	0.65	1.05	
		4th	853	60	7.0	0.75	0.55	1.02	0.75	0.55	1.02	0.7
	Physical	1st	1009	153	15.2	1			1			
		2nd-3rd	1 524	113	7.4	0.77	0.59	1	0.77	0.59	1.01	
		4th	1131	117	10.3	0.81	0.62	1.06	0.82	0.63	1.06	0.9
Mini-Finland	Mental	1st	181	25	13.8	1			1			
		2nd-3rd	415	29	7.00	0.78	0.45	1.34	0.77	0.45	1.34	
		4th	364	36	9.9	0.77	0.46	1.28	0.73	0.43	1.24	0.8
	Social	1st	308	23	7.5	1			1			
		2nd-3rd	443	43	9.7	1.18	0.71	1.96	1.15	0.69	1.92	
		4th	208	23	11.1	1.09	0.61	1.94	1.05	0.58	1.9	0.7
	Outdoor	1st	247	29	11.7	1			1			
		2nd-3rd	333	22	6.6	0.8	0.45	1.39	0.79	0.45	1.38	
		4th	376	38	10.1	0.85	0.52	1.38	0.85	0.52	1.38	0.3
	Consumptive	1st	110	10	9.1	1			1			
		2nd-3rd	593	60	10.1	1.17	0.60	2.28	1.14	0.58	2.23	
		4th	254	19	7.5	0.82	0.38	1.77	0.76	0.35	1.67	0.6
	Physical	1st	236	33	14.0	1			1			
		2nd-3rd	443	29	6.6	0.57	0.34	0.94	0.57	0.34	0.94	
		4th	374	39	10.4	0.64	0.4	1.04	0.65	0.4	1.06	0.5
HeSSup	Mental	1st	1233	3	0.2	1			1			
		2nd-3rd	3469	18	0.5	2.12	0.62	7.19	2.34	0.68	8.09	
		4th	1253	3	0.2	NA	0.20	4.83	1.17	0.23	6.04	0.9
	Social	1st	958	5	0.5	1						
		2nd-3rd	3257	12	0.4	0.70	0.25	1.99	0.73	0.26	2.08	
		4th	1781	8	0.5	0.85	0.28	2.60	0.88	0.29	2.71	0.6
	Outdoor	1st	958	5	0.5	1						
		2nd-3rd	3257	12	0.4	1.05	0.41	2.70	1.10	0.42	2.84	
		4th	1781	8	0.5	0.48	0.16	1.42	0.47	0.16	1.41	0.6
	Consumptive	1st	745	1	0.1	1						
		2nd-3rd	3667	17	0.5	3.44	0.46	25.86	3.73	0.49	28.20	
		4th	1562	6	0.4	2.86	0.34	23.74	3.28	0.39	27.69	0.8
	Physical	1st	1607	6	0.4	1						
		2nd-3rd	2967	14	0.5	1.24	0.48	3.23	1.26	0.48	3.30	
		4th	1510	4	0.3	0.70	0.20	2.46	0.70	0.20	2.48	0.6
FPS	Mental	1st	3574	32	0.9	1			1			
		2nd-3rd	10399	58	0.6	0.62	0.40	0.95	0.66	0.43	1.02	
		4th	5321	20	0.4	0.42	0.24	0.73	0.47	0.26	0.83	0.2
	Social	1st	4046	24	0.6	1			1			
		2nd-3rd	9523	61	0.6	1.08	0.67	1.73	1.03	0.64	1.65	
		4th	5791	27	0.5	0.78	0.45	1.36	0.65	0.37	1.13	0.2
	Outdoor	1st	2351	26	1.1	1			1			
		2nd-3rd	5462	29	0.5	0.47	0.28	0.80	0.51	0.30	0.87	
		4th	11427	54	0.5	0.42	0.26	0.67	0.44	0.27	0.70	0.3
	Consumptive	1st	5334	37	0.7	1			1			
		2nd-3rd	11429	58	0.5	0.73	0.48	1.10	0.70	0.46	1.07	
		4th	2530	16	0.6	0.91	0.50	1.63	0.81	0.44	1.48	0.5
	Physical	1st	4698	46	1.0	1			1			
		2nd-3rd	9979	52	0.5	0.53	0.35	0.78	0.57	0.38	0.85	
		4th	4713	14	0.3	0.30	0.16	0.54	0.37	0.20	0.68	0.8
SNAC-K	Mental	1st	754	164	21.8	1			1			
		2nd-3rd	1301	107	8.2	0.54	0.42	0.70	0.54	0.42	0.69	
		4th	685	27	3.9	0.38	0.25	0.59	0.37	0.24	0.57	0.9
	Social	1st	1019	186	18.3	1			1			
		2nd-3rd	1442	98	6.8	0.62	0.48	0.80	0.61	0.47	0.79	
		4th	279	14	5.0	0.48	0.28	0.83	0.47	0.27	0.83	0.1
	Outdoor	1st	747	163	21.8	1			1			
		2nd-3rd	1538	115	7.5	0.52	0.40	0.66	0.51	0.39	0.65	
		4th	455	20	4.4	0.38	0.23	0.60	0.39	0.24	0.62	0.5
	Consumptive	1st	710	157	22.1	1			1			
		2nd-3rd	1511	124	8.2	0.50	0.39	0.63	0.49	0.38	0.63	
		4th	519	17	3.3	0.35	0.21	0.58	0.36	0.21	0.60	<0.0001
	Physical	1st	699	144	20.6	1			1			
		2nd-3rd	1470	131	8.9	0.53	0.41	0.67	0.52	0.41	0.66	
		4th	571	23	4.0	0.34	0.21	0.53	0.33	0.21	0.52	0.2

Note: Quartile refers to the frequency of participation in each activity, divided into quartiles.
Hazard ratios are adjusted for age, sex and education.

Supplementary table 4. Study-specific associations of activities with the risk of dementia in ≥10 years' follow-up

Study	Activity	Quartile	N participants	N cases	% cases	Unadjusted HR	95% CI		Adjusted HR	95% CI		p for interaction with education
Health2000	Mental	1st	539	87	16.1	1			1			
		2nd-3rd	1416	175	12.4	0.68	0.53	0.88	0.70	0.53	0.92	
		4th	500	74	14.8	0.83	0.61	1.13	0.89	0.63	1.24	0.6
	Social	1st	651	102	15.7	1			1			
		2nd-3rd	1 282	161	12.6	0.70	0.55	0.89	0.71	0.56	0.92	
		4th	520	74	14.2	0.67	0.49	0.9	0.68	0.5	0.93	0.7
	Outdoor	1st	449	56	12.5	1			1			
		2nd-3rd	850	107	12.6	1.15	0.83	1.59	1.2	0.86	1.66	
		4th	1132	169	14.9	1.11	0.82	1.5	1.14	0.85	1.55	0.7
	Consumptive	1st	528	95	18.0	1			1			
		2nd-3rd	1235	159	12.9	0.81	0.63	1.04	0.84	0.65	1.08	
		4th	686	83	12.1	0.77	0.57	1.03	0.881	0.6	1.1	0.026
	Physical	1st	549	77	12.0	1			1			
		2nd-3rd	1 187	125	10.5	0.77	0.58	1.03	0.79	0.59	1.05	
		4th	794	141	17.8	1.07	0.8	1.42	1.08	0.81	1.44	0.9
HeSSup	Mental	1st	1173	7	0.6	1			1.00			
		2nd-3rd	3295	23	0.7	1.16	0.50	2.71	0.99	0.42	2.37	
		4th	1203	5	0.4	0.69	0.22	2.16	0.56	0.17	1.84	1.0
	Social	1st	898	5	0.6	1			1.00			
		2nd-3rd	3102	22	0.7	1.27	0.48	3.36	1.22	0.46	3.23	
		4th	1709	9	0.5	0.94	0.32	2.81	0.91	0.30	2.71	1.0
	Outdoor	1st	1310	10	0.8	1			1.00			
		2nd-3rd	1997	14	0.7	0.91	0.40	2.04	0.87	0.39	1.98	
		4th	2370	10	0.4	0.55	0.23	1.31	0.56	0.23	1.35	0.8
	Consumptive	1st	696	8	1.2	1			1.00			
		2nd-3rd	3498	21	0.6	0.52	0.23	1.17	0.49	0.22	1.12	
		4th	1492	6	0.4	0.35	0.12	0.99	0.32	0.11	0.94	0.9
	Physical	1st	1504	11	0.7	1			1.00			
		2nd-3rd	2834	16	0.6	0.77	0.36	1.65	0.72	0.33	1.55	
		4th	1448	8	0.6	0.75	0.30	1.87	0.75	0.30	1.87	0.5
SNAC-K	Mental	1st	195	13	6.7	1			1.00			
		2nd-3rd	771	46	6.0	1.69	0.89	3.19	1.73	0.91	3.28	
		4th	508	21	4.1	1.30	0.64	2.65	1.31	0.64	2.68	0.3
	Social	1st	327	22	6.7	1			1.00			
		2nd-3rd	957	50	5.2	1.52	0.90	2.58	1.54	0.90	2.62	
		4th	190	8	4.2	1.10	0.48	2.50	1.12	0.49	2.56	1.0
	Outdoor	1st	193	16	8.3	1			1.00			
		2nd-3rd	943	50	5.3	1.06	0.59	1.88	1.07	0.59	1.95	
		4th	338	14	4.1	1.07	0.51	2.23	1.10	0.51	2.37	1.0
	Consumptive	1st	1884	16	8.7	1			1.00			
		2nd-3rd	891	54	6.1	1.12	0.63	1.99	1.13	0.63	2.02	
		4th	399	10	2.5	0.70	0.31	1.60	0.71	0.31	1.64	0.5
	Physical	1st	195	18	9.2	1			1.00			
		2nd-3rd	854	45	5.3	0.85	0.49	1.48	0.86	0.49	1.51	
		4th	425	17	4.0	0.77	0.39	1.50	0.78	0.40	1.53	0.5

Note: Quartile refers to the frequency of participation in each activity, divided into quartiles.
Hazard ratios are adjusted for age, sex and education.

Supplementary table 5. Study-specific associations of variety of activities across domains with the risk of dementia, by follow-up period

		Follow-up <10 years										Follow-up >= 10 years									
Study	N domains	N participants	N cases	% cases	Unadjusted HR	95% CI		Adjusted HR	95% CI		p for interaction with education	N participants	N cases	% cases	Unadjusted HR	95% CI		Adjusted HR	95% CI		p for interaction with education
Health2000	0	1274	172	13.5	1			1				717	90	12.6	1			1			
	1	1057	103	9.7	0.79	0.61	1.02	0.79	0.61	1.02		741	95	12.8	0.84	0.63	1.12	0.86	0.63	1.15	
	2	846	76	9.0	0.69	0.52	0.91	0.69	0.52	0.91		634	84	13.3	0.85	0.63	1.15	0.87	0.65	1.18	
	>=3	600	49	8.2	0.67	0.48	0.92	0.66	0.48	0.92	0.2	474	82	17.3	1.02	0.75	1.38	1.08	0.79	1.46	0.3
Mini-Finland	0	298	28	9.4	1.00			1				NA									
	1	295	26	8.8	0.72	0.42	1.24	0.72	0.41	1.23											
	2	223	25	11.2	0.78	0.45	1.34	0.77	0.44	1.32											
	>=3	241	22	9.1	0.63	0.36	1.11	0.62	0.35	1.09	0.1										
HeSSup	0	1545	8	0.52	1			1				1449	10	0.69	1						
	1	2024	11	0.54	1.04	0.42	2.58	1.04	0.42	2.58		1915	16	0.84	1.21	0.55	2.66	1.22	0.55	2.68	
	2	1503	5	0.33	0.63	0.21	1.93	0.64	0.21	1.96		1438	8	0.56	0.80	0.32	2.03	0.82	0.32	2.09	
	>=3	1060	2	0.19	0.36	0.08	1.67	0.36	0.08	1.71	1.0	1026	2	0.19	0.28	0.06	1.27	0.28	0.06	1.29	1.0
FPS	0	4387	43	0.98	1			1				NA									
	1	6111	30	0.49	0.50	0.31	0.79	0.48	0.30	0.77											
	2	5043	31	0.61	0.62	0.39	0.98	0.63	0.39	1.00											
	>=3	4059	12	0.30	0.30	0.16	0.56	0.29	0.15	0.55	0.8										
SNAC-K	0	1391	235	16.90	1			1				519	39	7.50	1			1.00			
	1	632	38	6.00	0.54	0.38	0.77	0.54	0.38	0.77		408	19	4.70	0.97	0.56	1.70	0.97	0.55	1.70	
	2	397	15	3.80	0.41	0.24	0.69	0.40	0.24	0.69		292	15	5.10	1.12	0.61	2.06	1.12	0.61	2.06	
	>=3	320	10	3.10	0.40	0.21	0.76	0.41	0.22	0.79	<0.0001	255	7	2.80	0.63	0.28	1.43	0.64	0.28	1.45	<0.0001

Note: Hazard ratios are adjusted for age, sex and education.

Hazard ratios compare participants who were in the most active participation quartile (4th) in 1, 2 or ≥3 domains to those who were not in the most active participation quartile in any domain.

Supplementary table 6. Study-specific associations of the overall frequency of activities across domains with the risk of dementia

	Follow-up <10 years							Follow-up >=10 years					
**Study**	**Highest quartile**	**N participants**	**N cases**	**% cases**	**Adjusted HR**	**95% CI**		**N participants**	**N cases**	**% cases**	**Adjusted HR**	**95% CI**	
Health2000	1	251	58	23.1	1			59	9	15.3	1		
	2	1 023	114	11.1	0.65	0.45	0.93	658	81	12.3	1.09	0.51	2.29
	>=3	2503	228	9.1	0.52	0.45	0.73	1849	261	14.1	0.99	0.48	2.05
Mini-Finland	1	45	9	20.0	1.00			NA					
	2	253	19	7.5	0.66	0.29	1.48						
	>=3	759	73	9.6	0.52	0.26	1.05						
HeSSup	1	62	0	0.0	NA			55	1	1.8	1		
	2	1463	8	0.6				1374	9	0.7	0.31	0.04	2.45
	>=3	4548	15	0.4				4343	26	0.6	0.29	0.04	2.16
FPS	1	281	6	2.1	1			NA					
	2	4106	37	0.9	0.45	0.19	1.08						
	>=3	15213	73	0.5	0.23	0.10	0.54						
SNAC-K	1	303	90	29.7	1			30	10	30.0	1		
	2	1088	145	13.0	0.43	0.32	0.56	489	36	7.4	1.23	0.37	4.06
	>=3	1349	63	4.7	0.25	0.17	0.35	955	41	4.3	1.12	0.34	3.75

Note: Hazard ratios compare individuals with ≥3rd or 2nd as their highest activity participation quartile, across the domains, to those with 1st as their highest quartile.
Hazard ratios are adjusted for age, sex and education.

Supplementary table 7. Study-specific associations of activities with the risk of dementia, with additional adjustment for depression, mobility difficulties, sensory (vision and/or hearing) difficulties and characteristics of the municipality of residence

	Follow-up <10 years								Follow-up >=10 years					
**Study**	**Activity**	**Quartile**	**N participants**	**N cases**	**% cases**	**Adjusted HR**	**95% CI**		**N participants**	**N cases**	**% cases**	**Adjusted HR**	**95% CI**	
Health2000	Mental	1st	903	128	14.2	1			513	78	15.2	1		
		2nd-3rd	1787	140	7.8	0.65	0.50	0.85	1388	169	12.2	0.70	0.52	0.93
		4th	590	36	6.1	0.55	0.37	0.82	482	70	14.5	0.86	0.61	1.23
	Social	1st	929	111	12.0	1			618	94	15.2	1		
		2nd-3rd	1670	142	8.5	0.68	0.53	0.88	1255	153	12.2	0.69	0.53	0.89
		4th	678	51	7.5	0.45	0.32	0.64	510	71	13.9	0.66	0.47	0.91
	Outdoor	1st	785	98	12.5	1			428	53	12.4	1		
		2nd-3rd	1035	70	6.8	0.95	0.68	1.33	831	100	12.0	1.14	0.81	1.60
		4th	1404	124	8.8	0.99	0.73	1.33	1104	162	14.7	1.12	0.81	1.54
	Consumptive	1st	870	124	14.3	1			500	87	17.4	1		
		2nd-3rd	1563	123	7.9	0.79	0.61	1.04	1203	149	12.4	0.84	0.64	1.10
		4th	836	56	6.7	0.74	0.53	1.03	677	82	12.1	0.84	0.61	1.14
	Physical	1st	793	96	12.1	1			507	67	13.2	1		
		2nd-3rd	1426	102	7.2	0.91	0.68	1.23	1128	120	10.6	0.81	0.60	1.10
		4th	991	98	9.9	0.93	0.71	1.31	724	127	17.5	1.08	0.79	1.47
Mini-Finland	Mental	1st	171	21	12.3	1			NA					
		2nd-3rd	408	29	7.1	0.88	0.49	1.58						
		4th	362	36	9.9	0.79	0.45	1.39						
	Social	1st	303	21	6.9	1								
		2nd-3rd	433	42	9.7	1.20	0.71	2.04						
		4th	204	22	10.8	1.00	0.54	1.86						
	Outdoor	1st	236	27	11.4	1								
		2nd-3rd	330	21	6.4	0.81	0.45	1.45						
		4th	370	37	10.0	0.85	0.51	1.44						
	Consumptive	1st	101	8	7.9	1.00								
		2nd-3rd	584	58	9.9	1.14	0.54	2.42						
		4th	253	19	7.5	0.78	0.33	1.81						
	Physical	1st	196	24	12.2	1								
		2nd-3rd	423	29	6.9	0.55	0.32	0.96						
		4th	319	33	10.3	0.64	0.37	1.11						
HeSSup	Mental	1st	1144	3	0.3	1			1090	7	0.6	1.00		
		2nd-3rd	3209	16	0.5	2.10	0.60	7.38	3056	21	0.7	0.88	0.36	2.12
		4th	1163	3	0.3	1.15	0.22	5.99	1119	5	0.5	0.54	0.16	1.79
	Social	1st	876	4	0.5	1			825	5	0.6	1.00		
		2nd-3rd	3015	11	0.4	0.92	0.29	2.90	2876	21	0.7	1.18	0.44	3.15
		4th	1 666	8	0.5	1.28	0.38	4.31	1604	8	0.5	0.84	0.27	2.59
	Outdoor	1st	1272	6	0.5	1			1191	9	0.8	1.00		
		2nd-3rd	1935	10	0.5	1.16	0.42	3.22	1854	13	0.7	0.90	0.38	2.12
		4th	2310	6	0.3	0.57	0.18	1.79	2224	10	0.5	0.64	0.26	1.60
	Consumptive	1st	689	1	0.2	1			646	8	1.2	1.00		
		2nd-3rd	3432	15	0.4	3.31	0.43	25.27	3283	20	0.6	0.48	0.21	1.10
		4th	1416	6	0.4	3.36	0.40	28.54	1353	5	0.4	0.28	0.09	0.88
	Physical	1st	1469	5	0.3	1			1379	10	0.7	1.00		
		2nd-3rd	2 754	13	0.5	1.38	0.49	3.91	2639	15	0.6	0.74	0.33	1.65
		4th	1400	4	0.3	0.82	0.22	3.09	1346	8	0.6	0.84	0.33	2.14
FPS	Mental	1st	3446	28	0.8	1			NA					
		2nd-3rd	10124	54	0.5	0.76	0.48	1.21						
		4th	5183	17	0.3	0.53	0.28	0.99						
	Social	1st	3911	19	0.5	1								
		2nd-3rd	9258	56	0.6	1.30	0.77	2.20						
		4th	5 628	25	0.4	0.86	0.46	1.58						
	Outdoor	1st	2266	22	1.0	1								
		2nd-3rd	5311	27	0.5	0.61	0.35	1.09						
		4th	11127	50	0.5	0.57	0.33	0.97						
	Consumptive	1st	5156	32	0.6	1.00								
		2nd-3rd	11136	53	0.5	0.82	0.52	1.29						
		4th	2458	15	0.6	1.01	0.53	1.90						
	Physical	1st	4495	42	0.9	1								
		2nd-3rd	9 715	45	0.5	0.58	0.37	0.90						
		4th	4618	14	0.3	0.46	0.24	0.86						
SNAC-K	Mental	1st	675	148	21.9	1			185	12	6.5	1		
		2nd-3rd	1249	102	8.2	0.68	0.52	0.89	742	44	5.9	1.78	0.89	3.54
		4th	672	26	3.9	0.52	0.33	0.83	499	21	4.2	1.42	0.66	3.10
	Social	1st	931	168	18.1	1			312	21	6.7	1		
		2nd-3rd	1393	94	6.8	0.81	0.61	1.07	929	48	5.2	1.56	0.89	2.72
		4th	272	14	5.2	0.68	0.38	1.19	185	8	4.3	1.15	0.49	2.69
	Outdoor	1st	676	146	21.6	1			185	15	8.1	1		
		2nd-3rd	1475	111	7.5	0.68	0.51	0.89	909	48	5.3	1.10	0.59	2.05
		4th	445	19	4.3	0.57	0.34	0.95	332	14	4.2	1.19	0.54	2.65
	Consumptive	1st	646	141	21.8	1			178	16	9.0	1		
		2nd-3rd	1451	120	8.3	0.61	0.47	0.79	864	51	5.9	1.00	0.55	1.82
		4th	499	15	3.0	0.47	0.27	0.83	384	10	2.6	0.70	0.30	1.63
	Physical	1st	639	130	20.3	1			188	17	9.0	1		
		2nd-3rd	1406	124	8.8	0.65	0.50	0.85	824	43	5.2	0.86	0.47	1.56
		4th	551	22	4.0	0.46	0.28	0.74	414	17	4.1	0.77	0.38	1.59

Notes: Quartile refers to the frequency of participation in each activity, divided into quartiles.
Hazard ratios in HeSSup are adjusted for age, sex, depression and characteristics of the municipality of residence (urban, semi-urban, rural). Hazard ratios in Health 2000 and FPS are are adjusted for age, sex, education, depression, mobility difficulties, sensory difficulties and characteristics of the municipality of residence (urban, semi-urban, rural).
Hazard ratios in SNAC-K and Mini-Finland are are adjusted for age, sex, education, depression, mobility difficultiesa nd sensory difficulties; in these studies, all participants lived in urban areas.
Some participants had incomplete data on depresssion, mobility difficulties and/or sensory difficulties, and the numbers of participants included in the analyses adjusted for these covariates are smaller than the numbers of participants in our main analyses.

Supplementary table 8. Study-specific associations of activities with the risk of dementia among participants with no high-risk alleles on APOE gene

	Follow-up <10 years								Follow-up ≥ 10 years					
Study	Activity	Quartile	N participants	N cases	% cases	Adjusted HR	95% CI		N participants	N cases	% cases	Adjusted HR	95% CI	
Health2000	Mental activity	1st	583	74	12.7	1			337	41	12.2	1		
		2nd-3rd	1178	68	5.8	0.52	0.36	0.75	934	102	10.9	0.78	0.53	1.16
		4th	386	17	4.4	0.43	0.24	0.76	319	44	13.8	1.04	0.65	1.66
	Social activity	1st	601	59	9.8	1			414	54	13.00	1		
		2nd-3rd	1107	71	6.4	0.52	0.39	0.81	854	95	11.1	0.64	0.46	0.89
		4th	435	29	6.7	0.48	0.3	0.77	321	38	11.8	0.58	0.38	0.88
	Outdoor activity	1st	526	57	10.8	1			294	29	9.9	1		
		2nd-3rd	676	31	4.6	0.70	0.44	1.13	557	57	10.2	1.10	0.71	1.73
		4th	906	64	7.1	0.86	0.59	1.28	724	99	13.7	1.23	0.82	1.85
	Consumptive activity	1st	551	59	10.7	1			329	52	15.8	1		
		2nd-3rd	1050	71	6.8	0.95	0.66	1.37	818	92	11.3	0.90	0.64	1.28
		4th	538	29	5.4	0.84	0.52	1.35	440	43	9.8	0.79	0.52	1.19
	Physical activity	1st	511	51	10.0	1			339	42	21.4	1		
		2nd-3rd	935	55	5.9	0.91	0.6	1.38	749	68	9.1	0.73	0.5	1.08
		4th	647	47	7.3	0.88	0.58	1.34	483	75	15.5	1.04	0.71	1.53
SNAC-K	Mental activity	1st	483	89	18.4				140	10	7.1	1		
		2nd-3rd	865	57	6.6	0.57	0.41	0.81	541	25	4.6	1.52	0.7	3.28
		4th	473	16	3.4	0.46	0.26	0.88	356	7	2	0.65	0.24	1.79
	Social activity	1st	658	105	16.0				233	14	6	1		
		2nd-3rd	974	52	5.3	0.59	0.42	0.84	669	23	3.4	1.32	0.65	2.67
		4th	189	5	2.7	0.31	0.31	0.76	135	5	3.7	1.23	0.43	3.54
	Outdoor activity	1st	479	89	18.6				140	12	8.6	1		
		2nd-3rd	1035	61	5.9	0.51	0.36	0.72	661	26	3.9	0.80	0.39	1.65
		4th	307	12	3.9	0.46	0.46	0.85	236	4	1.7	0.52	0.16	1.67
	Consumptive activity	1st	448	81	18.1				131	13	9.9	1		
		2nd-3rd	1013	69	6.8	0.55	0.39	0.76	621	27	4.4	0.71	0.36	1.41
		4th	360	12	3.3	0.50	0.26	0.94	285	2	0.7	0.20	0.04	0.92
	Physical activity	1st	433	77	17.8				138	11	8	1		
		2nd-3rd	995	70	7.0	0.51	0.37	0.72	596	24	4	0.84	0.40	1.74
		4th	393	15	3.8	0.43	0.24	0.75	303	7	2.3	0.68	0.26	1.78

Note: Quartile refers to the frequency of participation in each activity, divided into quartiles. Hazard ratios are adjusted for age, sex and education. Genotype data were not available for all participants and the numbers of participants included in the analyses including genotype data are smaller than the numbers of participants in our main analyses.

Supplementary table 9. Study-specific associations of activities with the risk of dementia among participants with any high-risk allele on APOE gene

	Follow-up <10 years								Follow-up ≥ 10 years					
**Study**	**Activity**	**Quartile**	**N participants**	**N cases**	**% cases**	**Adjusted HR**	**95% CI**		**N participants**	**N cases**	**% cases**	**Adjusted HR**	**95% CI**	
Health2000	Mental activity	1st	264	44	16.7	1			157	34	21.7	1		
		2nd-3rd	512	64	12.5	0.94	0.62	1.43	385	62	16.1	0.77	0.50	1.20
		4th	171	18	10.5	0.90	0.50	1.61	136	23	16.9	0.83	0.47	1.46
	Social activity	1st	262	41	15.7	1			168	37	22.00	1		
		2nd-3rd	482	65	13.7	0.94	0.64	1.39	352	54	15.3	0.77	0.52	1.15
		4th	203	20	9.9	0.48	0.27	0.84	158	28	17.7	0.63	0.37	1.05
	Outdoor activity	1st	202	34	16.8	1			112	21	18.8	1		
		2nd-3rd	314	34	10.8	1.22	0.76	1.96	242	41	16.9	1.13	0.66	1.92
		4th	422	54	12.8	1.19	0.76	1.86	322	57	17.7	0.91	0.55	1.5
	Consumptive activity	1st	254	53	20.9	1			144	32	22.2	1		
		2nd-3rd	429	47	11.0	0.75	0.51	1.12	325	51	15.7	0.71	0.46	1.08
		4th	262	26	9.9	0.66	0.41	1.08	206	36	17.2	0.81	0.50	1.31
	Physical activity	1st	231	37	16.0	1			146	20	13.7	1		
		2nd-3rd	421	42	10.0	0.93	0.6	1.46	327	50	15.3	1.15	0.68	1.93
		4th	282	44	15.6	1.13	0.73	1.77	200	48	24	0.36	0.80	2.30
SNAC-K	Mental activity	1st	163	47	28.8	1			41	3	7.3	1		
		2nd-3rd	375	43	11.5	0.57	0.41	0.81	212	18	8.5	2.08	0.56	7.69
		4th	198	11	5.6	0.46	0.26	0.88	148	14	9.5	2.21	0.59	8.26
	Social activity	1st	238	50	21.0	1			81	8	9.9	1		
		2nd-3rd	420	42	10.0	0.59	0.42	0.84	270	25	9.3	1.94	0.79	4.8
		4th	78	9	11.5	0.31	0.31	0.76	50	2	4.0	0.72	0.15	3.49
	Outdoor activity	1st	163	45	27.6	1			43	3	7.0	1		
		2nd-3rd	440	48	10.9	0.51	0.36	0.72	261	22	8.4	2.33	0.65	8.38
		4th	133	8	6.0	0.46	0.46	0.85	97	10	10.3	2.95	0.72	12.08
	Consumptive activity	1st	166	49	29.5	1			44	2	4.6	1		
		2nd-3rd	427	47	11.0	0.55	0.39	0.76	248	26	10.5	4.07	0.88	18.82
		4th	143	5	3.5	0.5	0.26	0.94	109	7	6.4	2.89	0.51	16.24
	Physical activity	1st	172	39	22.7	1			51	7	13.7	1		
		2nd-3rd	406	54	13.3	0.51	0.37	0.72	238	18	7.6	0.81	0.32	2.05
		4th	158	8	5.1	0.43	0.24	0.75	112	10	8.9	0.95	0.35	2.56

Note: Quartile refers to the frequency of participation in each activity, divided into quartiles. Hazard ratios are adjusted for age, sex and education. Genotype data were not available for all participants and the numbers of participants included in the analyses including genotype data are smaller than the numbers of participants in our main analyses.

Supplementary table 10. Associations of activities with the risk of dementia, using an alternative grouping of activities in SNAC-K (n=2,740)

Activity	Quartile	Follow-up <10 years<br>N participants	N (%) dementia	HR[1] (95% CI)	Follow-up ≥ 10 years<br>N participants	N (%) dementia	HR[1] (95% CI)
Mental activity	1st	781	155 (19.9)	1	225	17 (7.6)	1
	2nd-3rd	1,404	120 (8.6)	0.58 (0.46 to 0.74)	840	46 (5.5)	1.13 (0.64 to 2.00)
	4th	555	23 (4.4)	0.35 (0.22 to 0.54)	409	17 (4.2)	0.88 (0.44 to 1.76)
Social activity	1st	810	175 (21.6)	1	214	23 (10.8)	1
	2nd-3rd	1,424	114 (8.0)	0.52 (0.40 to 0.66)	865	46 (5.3)	0.78 (0.46 to 1.33)
	4th	506	9 (1.8)	0.22 (0.11 to 0.44)	395	11 (2.8)	0.68 (0.31 to 1.49)
Physical activity	1st	739	169 (22.9)	1	170	18 (10.6)	1
	2nd-3rd	1,534	117 (7.6)	0.48 (0.38 to 0.62)	928	50 (5.4)	0.88 (0.50 to 1.56)
	4th	467	12 (2.6)	0.29 (0.16 to 0.53)	376	12 (3.2)	0.76 (0.35 to 1.64)
**Number of domains**	0	1,756	262 (14.9)	1	730	52 (7.2)	1
	1	566	30 (1.4)	0.52 (0.32 to 0.76)	413	18 (4.4)	0.85 (0.49 to 1.47)
	2	292	<5	NA	226	8 (3.5)	0.78 (0.36 to 1.66)
	≥3	126	<5	NA		<5	NA
**Highest quartile**	1	432	122 (28.2)	1	67	11 (16.4)	1
	2	1,324	140 (10.6)	0.45 (0.35 to 0.57)	663	41 (6.2)	0.58 (0.29 to 1.15)
	≥3	984	36 (3.7)	0.24 (0.16 to 0.36)	744	28 (3.8)	0.51 (0.24 to 1.07)

Notes:
Hazard ratios for the number of domains (variety of activities) compare participants who were in the most active participation quartile (4th) in 1, 2 or ≥3 domains to those who were not in the most active participation quartile in any domain.
Hazard ratios for the highest quartile (overall frequency of activity) compare individuals with ≥3rd or 2nd as their highest activity participation quartile, across the domains, to those with 1st as their highest quartile.
Hazard ratios are adjusted for age, sex and education.

Supplementary table 11. Study-specific association of domain-specific activity with the risk of dementia, excluding participants aged <60 years at baseline

			Follow-up <10 years						Follow-up >= 10 years					
Study	Actitivity	Quartile	N participants	N cases	% cases	Adjusted HR	95% CI		N participants	N cases	% cases	Adjusted HR	95% CI	
Health2000	Mental	1st	734	159	21.0	1.00			264	71	26.9	1.00		
		4th	351	41	11.7	0.55	0.38	0.79	238	60	25.2	0.87	0.60	0.96
	Social	1st	654	131	20.0	1.00			280	80	28.6	1.00		
		4th	466	57	12.2	0.47	0.34	0.66	292	62	21.2	0.64	0.46	0.90
	Outdoor	1st	624	120	19.2	1.00			206	47	22.8	1.00		
		4th	906	133	14.7	0.97	0.75	1.27	596	139	23.2	1.11	0.80	1.80
	Consumptive	1st	729	150	20.6	1.00			282	81	28.7	1.00		
		4th	431	54	12.5	0.72	0.52	0.99	287	70	24.4	0.86	0.62	1.19
	Physical	1st	657	149	22.7	1.00			240	62	25.8	1.00		
		4th	766	114	14.9	0.83	0.64	1.08	466	125	26.8	1.13	0.83	1.55
Mini-Finland	Mental	1st	128	25	19.5	1.00								
		4th	240	33	13.8	0.67	0.39	1.15						
	Social	1st	177	22	12.4	1.00								
		4th	142	22	15.5	1.04	0.57	1.90						
	Outdoor	1st	157	27	17.2	1.00								
		4th	259	38	14.7	0.92	0.55	1.51						
	Consumptive	1st	76	10	13.2	1.00								
		4th	157	18	11.5	0.73	0.33	1.61						
	Physical	1st	145	31	21.4	1.00								
		4th	263	38	14.5	0.68	0.41	1.11						
FPS	Mental	1st	2706	31	1.15	1.00								
		4th	4084	19	0.47	0.44	0.25	0.80						
	Social	1st	2754	23	0.84	1.00								
		4th	4941	27	0.55	0.63	0.36	1.11						
	Outdoor	1st	1754	26	1.48	1.00								
		4th	8941	53	0.59	0.42	0.26	0.67						
	Consumptive	1st	3735	36	0.96	1.00								
		4th	2145	15	0.70	0.72	0.39	1.34						
	Physical	1st	3675	44	1.20	1.00								
		4th	3349	14	0.42	0.39	0.21	0.71						
SNAC-K	Mental	1st	754	164	21.8	1.00			195	13	6.7	1.00		
		4th	685	27	3.9	0.37	0.24	0.57	508	21	4.1	1.31	0.64	2.68
	Social	1st	1019	186	18.3	1.00			327	22	6.7	1.00		
		4th	279	14	5.0	0.48	0.27	0.83	190	8	4.2	1.12	0.49	2.56
	Outdoor	1st	747	163	21.8	1.00			193	16	8.3	1.00		
		4th	455	20	4.4	0.38	0.24	0.62	338	14	4.1	1.10	0.51	2.36
	Consumptive	1st	710	157	22.1	1.00			184	16	8.7	1.00		
		4th	519	17	3.3	0.36	0.21	0.60	399	10	2.5	0.71	0.31	1.64
	Physical	1st	699	144	20.6	1.00			195	18	9.2	1.00		
		4th	571	23	4.0	0.33	0.21	0.52	425	17	4.0	0.78	0.4	1.53

Note: Quartile refers to the frequency of participation in each activity, divided into quartiles.
Hazard ratios are adjusted for age, sex and education.

Supplementary table 12. Study-specific associations of variety of activities across the domains the risk of dementia, excluding participants aged <60 years at baseline

		Follow-up <10 years						Follow-up >= 10 years					
**Study**	**N domains**	**N participants**	**N cases**	**% cases**	**Adjusted HR**	**95% CI**		**N participants**	**N cases**	**% cases**	**Adjusted HR**	**95% CI**	
Health2000	0	787	167	21.1	1.00			286	72	25.2	1.00		
	1	627	101	16.1	0.80	0.62	1.04	348	80	23.0	0.88	0.64	1.22
	2	492	74	15.0	0.69	0.52	0.92	305	64	21.0	0.79	0.56	1.10
	>=3	384	45	11.7	0.63	0.45	0.88	268	73	27.2	1.09	0.78	1.51
Mini-Finland	0	162	28	17.3	1.00								
	1	169	25	14.8	0.68	0.39	1.18						
	2	150	24	16.0	0.72	0.42	1.25						
	>=3	169	21	12.4	0.58	0.33	1.03						
FPS	0	3195	39	1.22	1.00								
	1	4681	30	0.64	0.52	0.32	0.84						
	2	3859	31	0.80	0.67	0.42	1.09						
	>=3	3286	11	0.33	0.28	0.14	0.55						
SNAC-K	0	1391	235	16.9	1.00			519	39	7.5	1.00		
	1	632	38	6.0	0.54	0.38	0.77	408	19	4.7	0.97	0.55	1.70
	2	397	15	3.8	0.40	0.24	0.69	292	15	5.1	1.12	0.61	2.06
	>=3	320	10	3.1	0.41	0.22	0.79	255	7	2.8	0.63	0.28	1.45

Note: Hazard ratios are adjusted for age, sex and education.
Hazard ratios compare participants who were in the most active participation quartile (4th) in 1, 2 or ≥3 domains to those who were not in the most active participation quartile in any domain.

Supplementary table 13. Study-specific association of the overall frequency of activities across the domains the risk of dementia, excluding participants aged <60 years at baseline

		Follow-up <10 years						Follow-up >=10 years					
**Study**	**Highest quartile**	**N participants**	**N cases**	**% cases**	**Adjusted HR**	**95% CI**		**N participants**	**N cases**	**% cases**	**Adjusted HR**	**95% CI**	
Health2000	1	212	56	26.4	1.00			29	9	31.0	1.00		
	2	575	111	19.3	0.70	0.49	1.01	257	63	24.5	0.87	0.41	1.87
	>=3	1503	220	14.6	0.55	0.39	0.77	921	217	23.6	0.80	0.38	1.67
Mini-Finland	1	34	9	26.5	1.00								
	2	128	19	14.8	0.68	0.30	1.54						
	>=3	488	70	14.3	0.50	0.25	1.03						
FPS	1	214	6	2.80	1.00								
	2	2981	33	1.11	0.40	0.17	0.97						
	>=3	11826	72	0.61	0.22	0.10	0.52						
SNAC-K	1	303	90	29.7	1.00			30	3	10.0	1.00		
	2	1088	145	44.9	0.43	0.32	0.56	489	36	7.4	1.23	0.37	4.06
	>=3	1349	63	4.7	0.25	0.17	0.35	955	41	4.3	1.12	0.34	3.75

Note: Hazard ratios compare individuals with ≥3rd or 2nd as their highest activity participation quartile, across the domains, to those with 1st as their highest quartile.
Hazard ratios are adjusted for age, sex and education.

Supplementary figure 1. Random effects meta-analyses of the association of domain-specific activity with the risk of dementia, by follow-up period

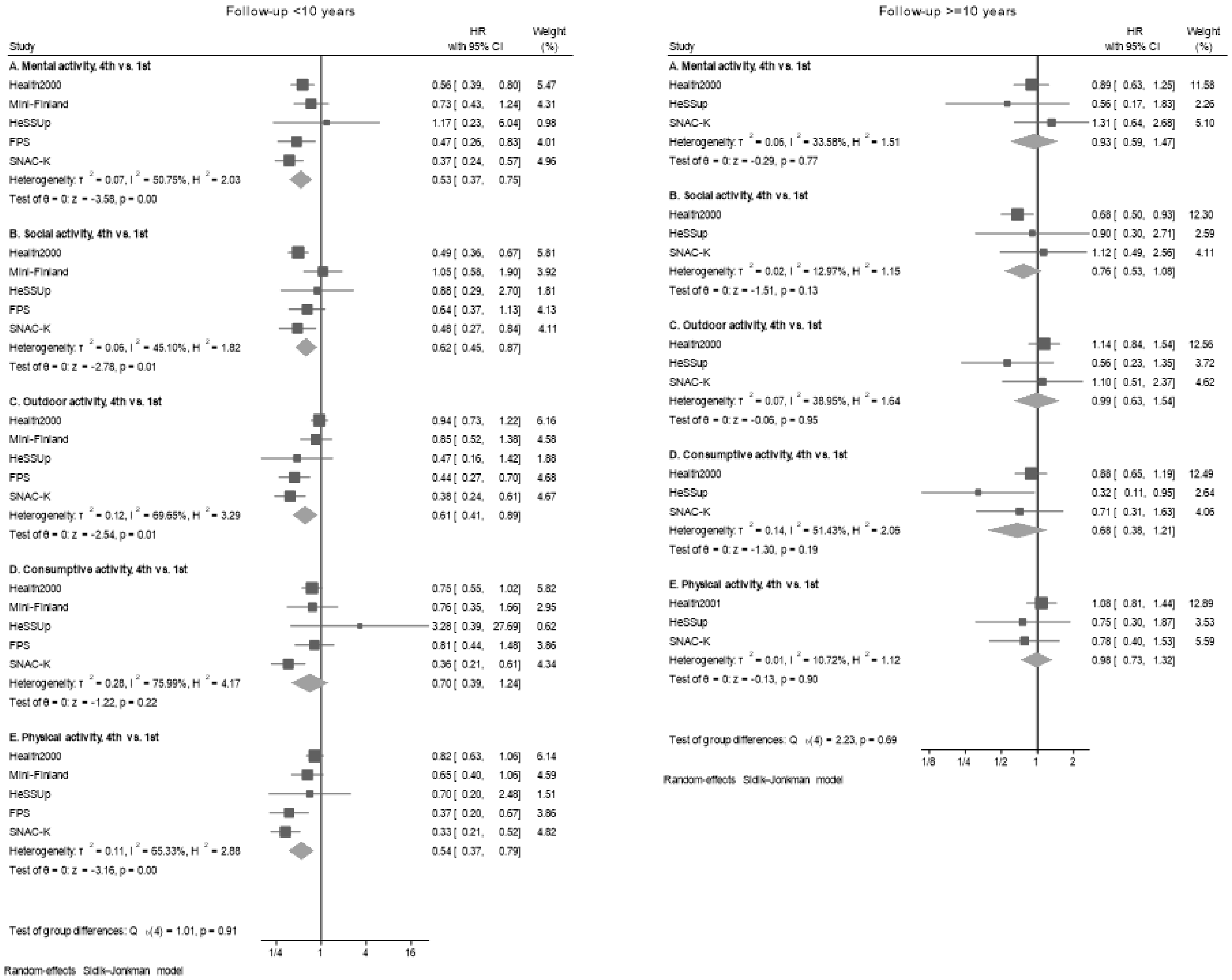

Note to supplementary figure 1: Hazard ratios are adjusted for age, sex and education. Hazard ratios compare the 4th (most active) to the 1st (least active) quartile of activity participation in each activity domain. Study-specific numbers of participants and dementia cases are presented in supplementary tables 3 and 4.

Supplementary figure 2. Random effects meta-analyses of the association of variety of activities across the domains with the risk of dementia, by follow-up period

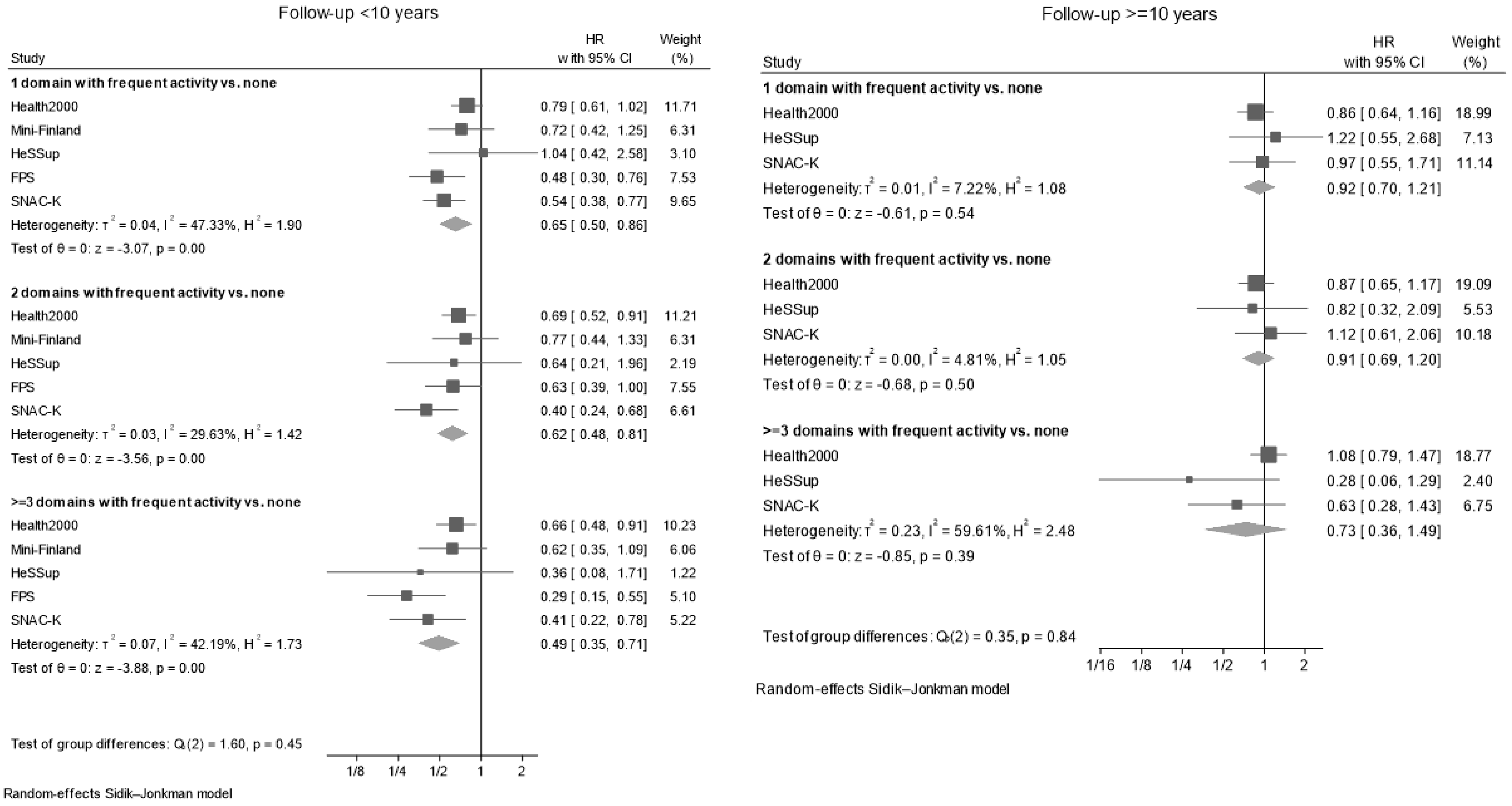

Note to supplementary figure 2. Adjusted for age, sex and education. Hazard ratios compare participants who were in the most active participation quartile (4th) in 1, 2 or ≥3 domains to those who were not in the most active participation quartile in any domain. Study-specific numbers of participants and dementia cases are presented in supplementary table 5.

Supplementary figure 3. Random effects meta-analyses of the association of frequency of activity across the domains with the risk of dementia, by follow-up period

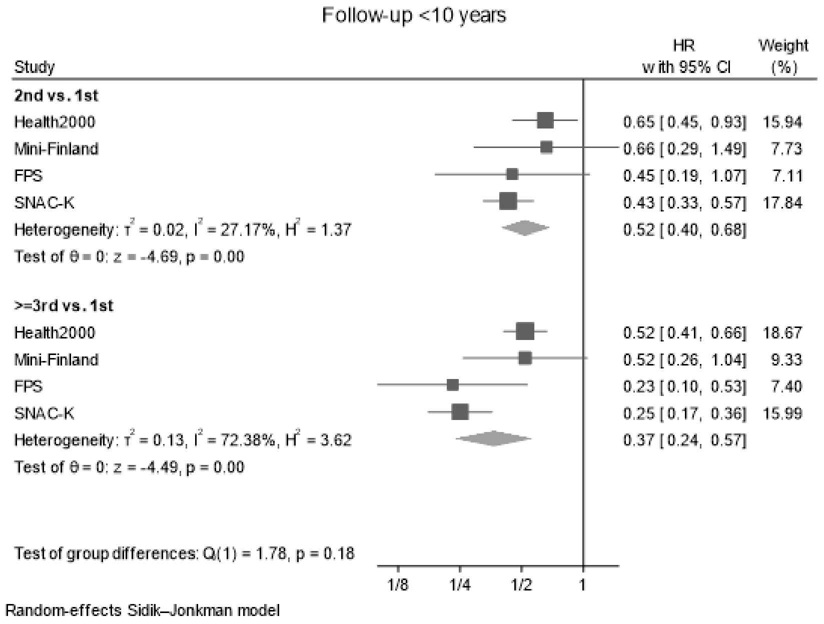

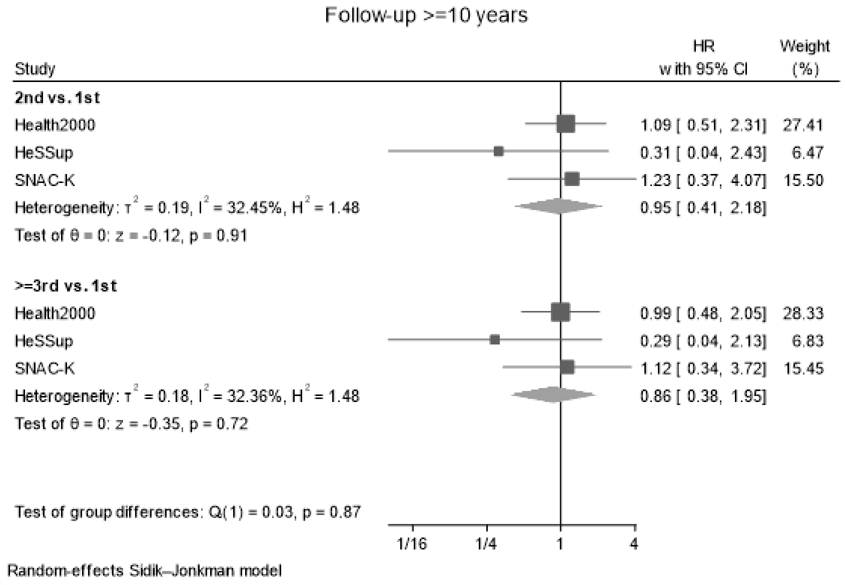

Note to supplementary figure 3: Adjusted for age, sex and education. HRs compare individuals with ≥3rd or 2nd as their highest activity participation quartile, across the domains, to those with 1st as their highest quartile. Study-specific numbers of participants and dementia cases are presented in supplementary table 6.

Supplementary figure 4. Fixed effects meta-analyses of the association of domain-specific activity with the risk of dementia, excluding participants aged <60 years at baseline

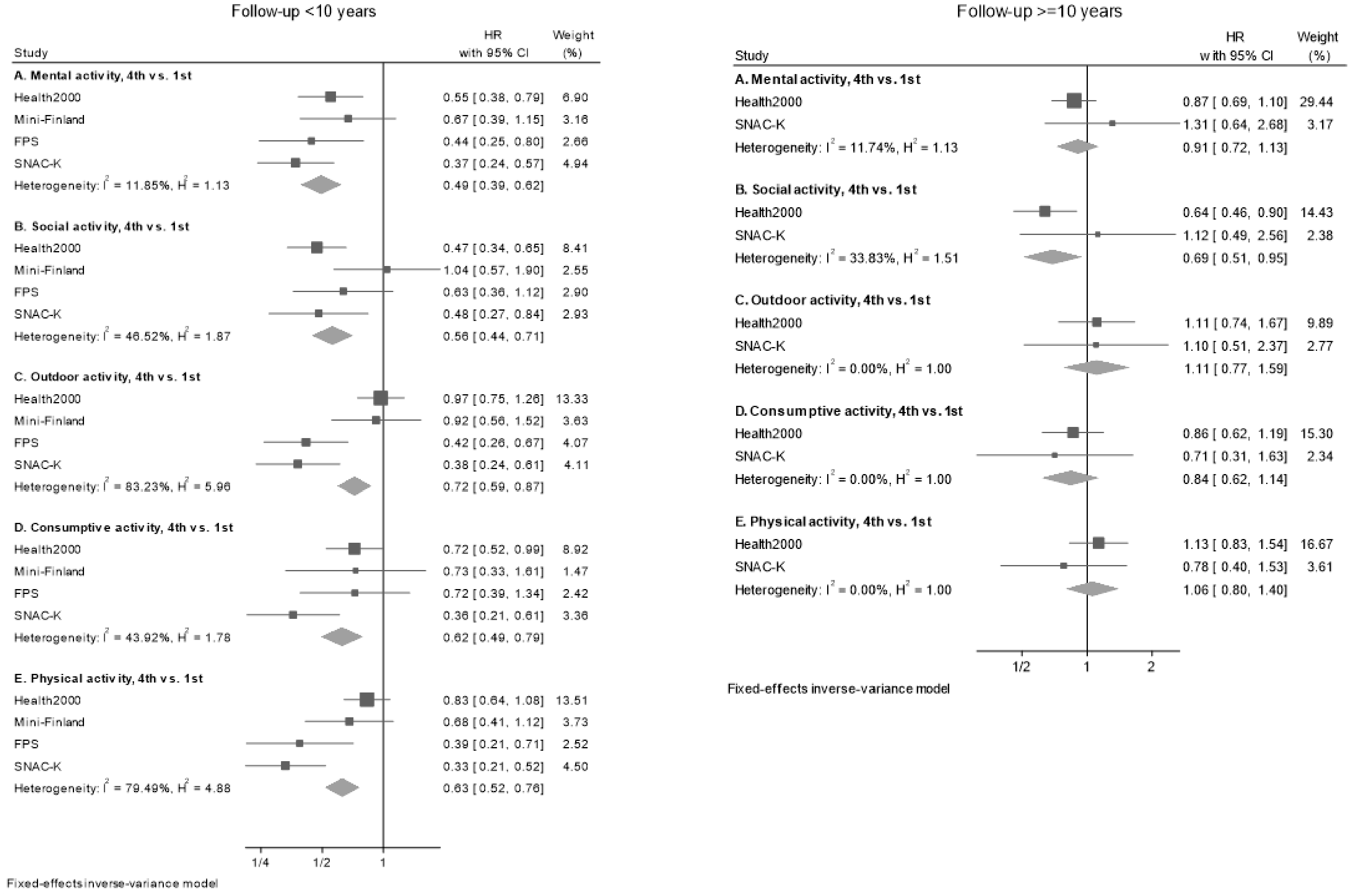

Note to supplementary figure 4: Hazard ratios are adjusted for age, sex and education and they compare the 4th (most active) to the 1st (least active) quartile of activity participation in each activity domain. Study-specific numbers of participants and dementia cases are presented in supplementary table 11.

Supplementary figure 5. Fixed effects meta-analyses of the association of variety of activities across the domains the risk of dementia, excluding participants aged <60 years at baseline

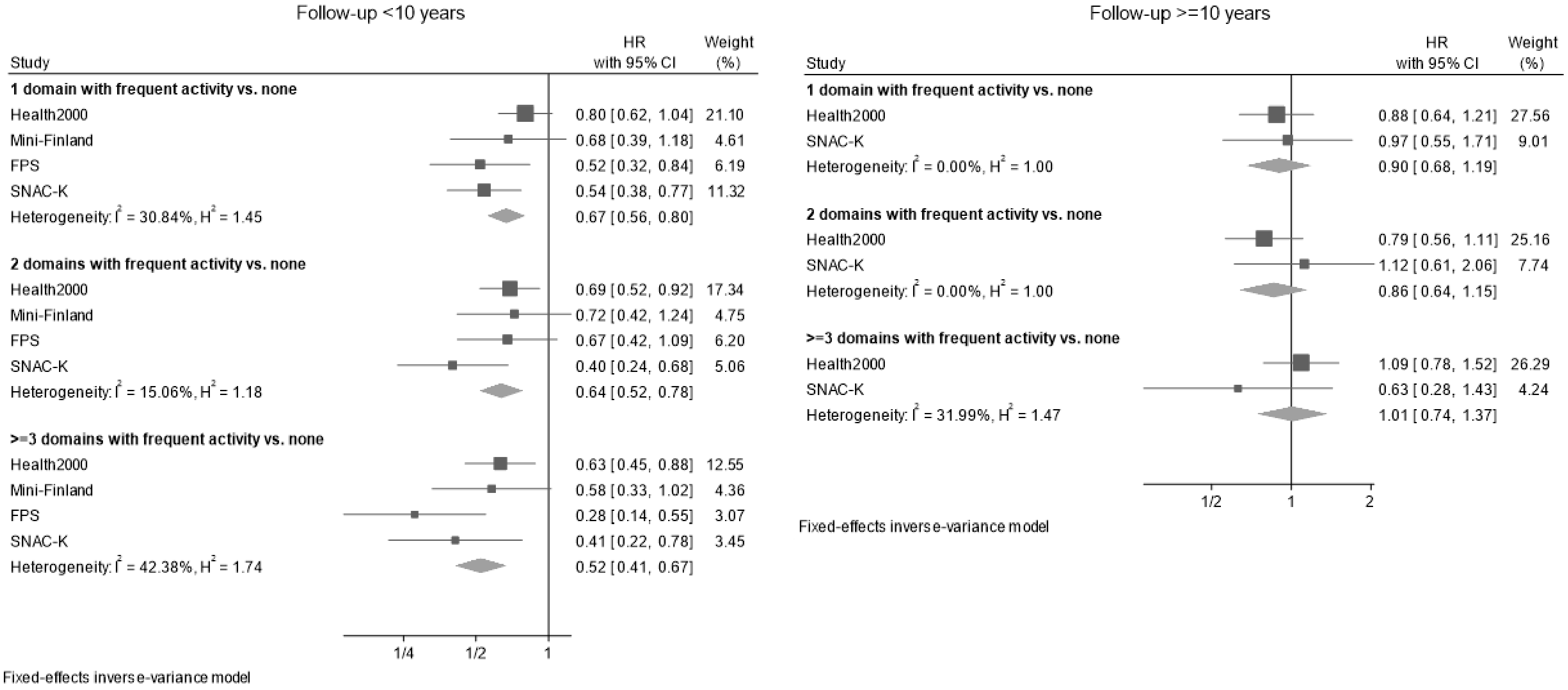

Note to supplementary figure 5. Adjusted for age, sex and education. Hazard ratios compare participants who were in the most active participation quartile (4th) in 1, 2 or ≥3 domains to those who were not in the most active participation quartile in any domain. Study-specific numbers of participants and dementia cases are presented in supplementary table 12.

Supplementary figure 6. Fixed effects meta-analyses of the association of the overall frequency of activity across the domains with the risk of dementia, excluding participants aged <60 years at baseline

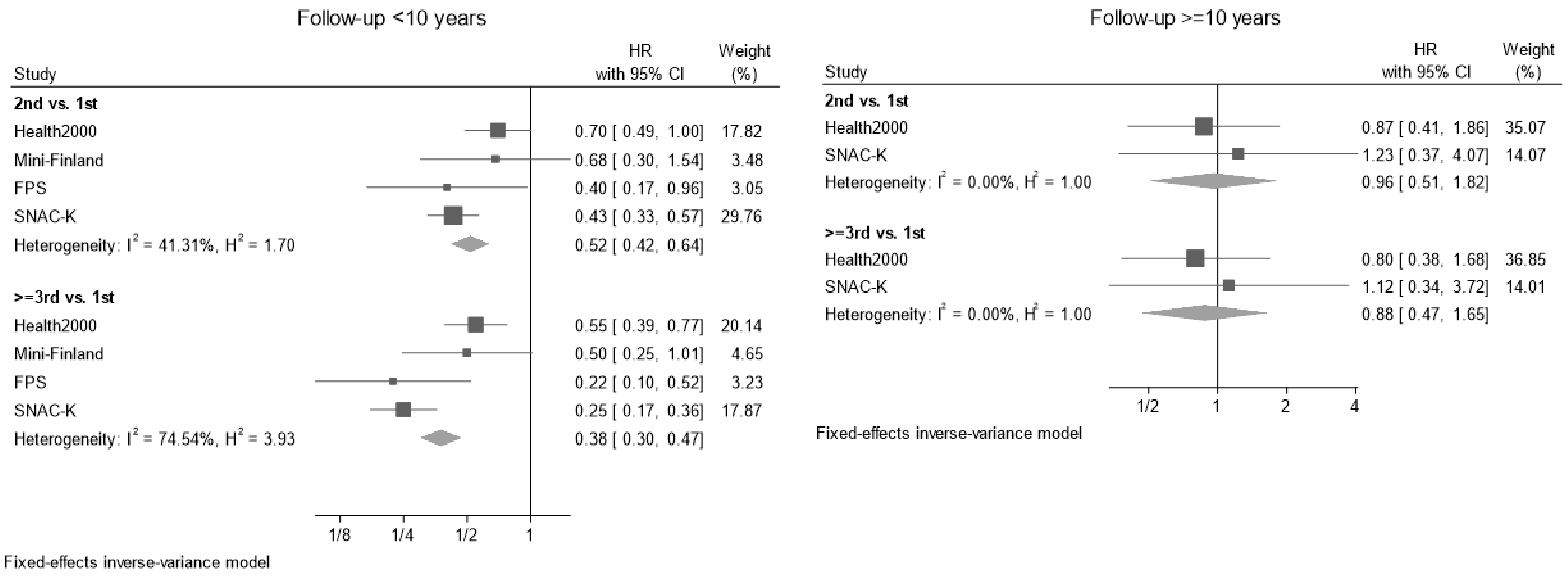

Note to supplementary figure 6: Adjusted for age, sex and education. HRs compare individuals with ≥3rd or 2nd as their highest activity participation quartile, across the domains, to those with 1st as their highest quartile. Study-specific numbers of participants and dementia cases are presented in supplementary table 13.

Supplementary figure 7. Fixed effects meta-analyses of the association of A) domain-specific activity, B) variety of activity and C) overall frequency of activity with the risk of dementia in <10 years follow-up in studies that had ≥10 years' follow-up

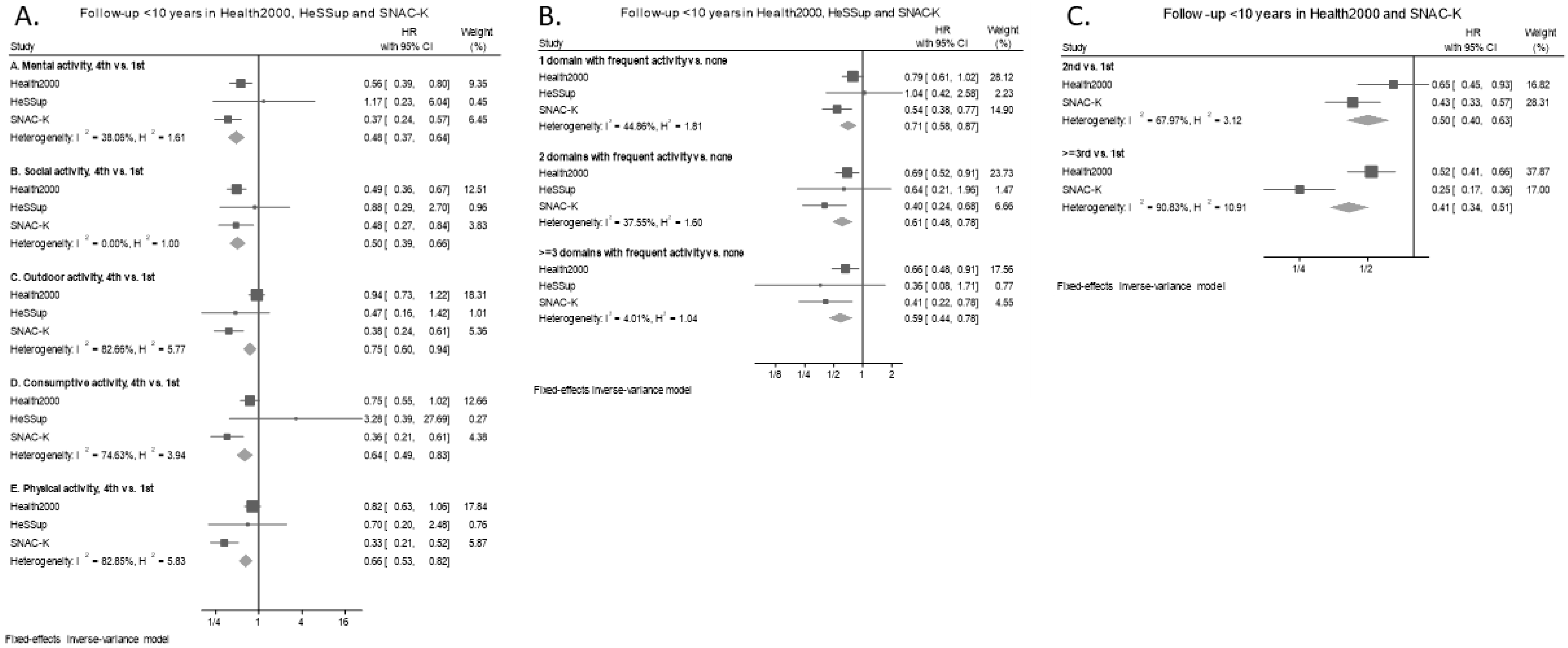

Appendix 3. Supplementary references

1. Korkeila K, Suominen S, Ahvenainen J, et al. Non-response and related factors in a nation-wide health survey. *Eur J Epidemiol* 2001;17(11):991-9.
2. Aromaa A KS. Health and Functional Capacity in Finland: Baseline Results of the Health 2000 Health Examination Survey: National Public Health Institute, 2004.
3. Heistaro Se. Methodology Report Health 2000 Survey. Helsinki: National Public Health Institute, 2008.
4. Heliovaara M, Aromaa A, Klaukka T, et al. Reliability and validity of interview data on chronic diseases. The Mini-Finland Health Survey. *J Clin Epidemiol* 1993;46(2):181-91.
5. Lundqvist A Mäki-Opas T. Health 2011 Survey -Methods: Finnish Institute for Health and Welfare, 2016.
6. Lagergren M, Fratiglioni L, Hallberg IR, et al. A longitudinal study integrating population, care and social services data. The Swedish National study on Aging and Care (SNAC). *Aging Clin Exp Res* 2004;16(2):158-68.
7. Kivimaki M, Lawlor DA, Davey Smith G, et al. Socioeconomic position, co-occurrence of behavior-related risk factors, and coronary heart disease: the Finnish Public Sector study. *Am J Public Health* 2007;97(5):874-9.
8. Sund R. Quality of the Finnish Hospital Discharge Register: a systematic review. *Scand J Public Health* 2012;40(6):505-15. doi: 10.1177/1403494812456637
9. Fratiglioni L, Viitanen M, vonStrauss E, et al. Very old women at highest risk of dementia and Alzheimer's disease: Incidence data from the Kungsholmen project, Stockholm. *Neurology* 1997;48(1):132-38.
10. Beck AT, Ward CH, Mendelson M, et al. An inventory for measuring depression. *Arch Gen Psychiatry* 1961;4:561-71. doi: 10.1001/archpsyc.1961.01710120031004
11. SA M, M A. A new depression scale designed to be sensitive to change. *Br J Psych* 1979;134(4):382-89. doi: doi:10.1192/bjp.134.4.382
12. Guralnik JM, Ferrucci L, Simonsick EM, et al. Lower-Extremity Function in Persons over the Age of 70 Years as a Predictor of Subsequent Disability. *New Engl J Med* 1995;332(9):556-61. doi: Doi 10.1056/Nejm199503023320902
13. Verghese PB, Castellano JM, Holtzman DM. Apolipoprotein E in Alzheimer's disease and other neurological disorders. *Lancet Neurol* 2011;10(3):241-52. doi: 10.1016/S1474-4422(10)70325-2 [published Online First: 2011/02/26]
14. Delaneau O, Zagury JF, Marchini J. Improved whole-chromosome phasing for disease and population genetic studies. *Nat Methods* 2013;10(1):5-6.
15. Howie B, Fuchsberger C, Stephens M, et al. Fast and accurate genotype imputation in genome-wide association studies through pre-phasing. *Nat Genet* 2012;44(8):955-+. doi: 10.1038/ng.2354
16. Loh PR, Danecek P, Palamara PF, et al. Reference-based phasing using the Haplotype Reference Consortium panel. *Nat Genet* 2016;48(11):1443-48.
17. Dekhtyar S, Marseglia A, Xu W, et al. Genetic risk of dementia mitigated by cognitive reserve: A cohort study. *Ann Neurol* 2019;86(1):68-78.
18. Thiebaut AC, Benichou J. Choice of time-scale in Cox's model analysis of epidemiologic cohort data: a simulation study. *Stat Med* 2004;23(24):3803-20.
19. Floud S, Balkwill A, Sweetland S, et al. Cognitive and social activities and long-term dementia risk: the prospective UK Million Women Study. *Lancet Public Health* 2021;6(2):e116-e23.
20. Felsch M, Beckmann L, Bender R, et al. Performance of several types of beta-binomial models in comparison to standard approaches for meta-analyses with very few studies. *BMC Med Res Methodol* 22, 319 (2022). https://doi.org/10.1186/s12874-022-01779-3
21. Rice K, Higgins JPT, Lumley T. A re-evaluation of fixed effect(s) meta-analysis. *J Roy Stat Soc A* 2018;181(1):205-27. doi: 10.1111/rssa.12275
22. Marseglia A, Wang HX, Rizzuto D, et al. Participating in Mental, Social, and Physical Leisure Activities and Having a Rich Social Network Reduce the Incidence of Diabetes-Related Dementia in a Cohort of Swedish Older Adults. *Diabetes Care* 2019;42(2):232-39.
23. Wang HX, Karp A, Winblad B, et al. Late-life engagement in social and leisure activities is associated with a decreased risk of dementia: A longitudinal study from the kungsholmen project. *Am J Epidemiol* 2002;155(12):1081-87.
